# Assembling an Expanded Metabolome for Camu-Camu (Myrciaria Dubia): Bioactive and Nutritional Compounds Relevant to Functional Food Research

**DOI:** 10.3390/molecules31183167

**Published:** 2026-09-09

**Authors:** Subramanyam Ragupathy, Varathan Vinayagam, Velautham Saravanan, Arunachalam Thirugnanasambandam, Steven G. Newmaster

**Affiliations:** 1 College of Biological Science, University of Guelph, Guelph, ON N1G 2W1, Canada; ragu@uoguelph.ca (S.R.); vinaychemist89@gmail.com (V.V.); saravananorganic2014@gmail.com (V.S.); athiru@outlook.com (A.T.); 2 Biological and Life Sciences, Canadian Light Source, Saskatoon, SK S7N 2V3, Canada

**Keywords:** camu-camu, *Myrciaria dubia*, metabolomics, ^1^H NMR, LC-MS, solvent extraction, functional foods, phytochemicals

## Abstract

Camu-camu (*Myrciaria dubia*) is an Amazonian shrub associated with riverine ecosystems and is recognized for its high vitamin C content and a broad range of nutritional and specialized metabolites. Previous studies have focused on specific metabolites or bioactive metabolites associated with health issues. Therefore, a comprehensive metabolomic survey using multiple analytical methods islacking. To address this gap in the scientific literature, we prepared a focused metabolomic inventory by integrating curated literature records with proton nuclear magnetic resonance (^1^H NMR) and liquid chromatography–mass spectrometry (LC-MS) observations from berry materials, including pulp, skin/peel, and seeds. Ourstudy added 107 metabolite annotation records that were not in the literature. The integration of the literature-derived and ourdatasets yielded a combined inventory of 260 metabolites. Seven solvents were compared using ^1^H NMR spectroscopy, and CH_3_OH:CD_3_OD (9:1, *v*/*v*) provided the broadest assigned coverage. LC-MS expanded the detection of lower-abundance and structurally diverse features, particularly phospholipids, quaternary ammonium compounds, organic acids, amino acid derivatives, and related metabolites. Compound-level and class-level assignments were distinguished, and interpretations were limited to the confidence supported by the available analytical evidence. The resulting inventory provides an expanded foundation for camu-camu quality evaluation, solvent selection, clinical trials, and targeted functional food research.

## 1. Introduction

Camu-camu [*Myrciaria dubia* (Kunth) McVaugh] is a shrub or small tree of the Myrtaceae family that is commonly found throughout the northern Amazon. It grows along riverbanks, floodplain lakes, and swampy ecosystems, where plants are partially immersed in water [1,2]. The fruit is spherical, grape-sized, and light orange to reddish-purple, with acidic yellowish pulp that becomes gray when dry and commonly contains one to four elliptical and flattened seeds [1]. This species is distributed across much of the Amazon region, including Brazil, Guyana, Venezuela, Bolivia, Colombia, Ecuador and Peru [3,4,5,6]. Considerable intraspecific diversity has been reported in Northern Amazon and Peruvian Amazon populations, including isolated or small populations, which is relevant for germplasm conservation, breeding, and future agricultural development [7,8,9,10]. Although the fruit is known locally by names such as caçari, araçá d’água, sarão, camocamo, or camu-camu, documented traditional use remains comparatively limited, with one ethnobotanical record from Peru reporting its use along the Nanay River for malaria-related applications [11,12,13].

Camu-camu fruits are frequently described as superfruits because they contain high concentrations of ascorbic acid and other bioactive constituents. Duke’s early phytochemical compilation and nutritional reviews identified amino acids and health-promoting phytochemicals in camu-camu [14,15], while bioactive compound and composition studies reported the presence of phenolic compounds, carotenoids, organic acids, and other specialized metabolites [16,17,18,19,20]. The best-known nutritional claim is the high vitamin C content reported in the pulp and fruit products. Published values vary substantially with genotype, maturity stage, tissue fraction, post-harvest handling, processing, and analytical methods. Selected reports cited in the camu-camu literature include approximately 1910.31 mg/100 g in green fruits and 2061.01 mg/100 g in purple fruits, while broader literature summaries report approximately 850–5000 mg/100 g of the edible portion in some materials [15,21,22,23]. Therefore, comparisons with other vitamin C-rich fruits should be treated as literature-context ranges rather than fixed compositional constants. Acerola (*Malpighia emarginata*) is widely recognized as a vitamin-C-rich fruit, and Kakadu plum (*Terminalia ferdinandiana*) has been reported to contain approximately 2300–3150 mg/100 g wet weight, and occasionally higher values [24,25,26]. Antioxidant compounds and antioxidant capacity also contribute to the functional food narrative surrounding camu-camu, especially phenolics, anthocyanins, ellagitannins, and related compounds reported in fruit, peel, and seed fractions [27,28,29].

Previous research has focused on the bioactive metabolites of camu-camu, which may have health benefits. The seed and peel fractions contain ellagitannins, acylphloroglucinols, phenolic acids, and other compounds with reported in vitro or preclinical activities. Methylvescalagin and seed-derived compounds have been associated with antiplasmodial, anti-hemolytic, antimicrobial, antihyperglycemic, anti-inflammatory, and antihypertensive activities in specific experimental contexts [30,31,32]. Recent reviews and model-based studies have discussed nutritional, pharmacological, industrial, oxidative stress, and functional food relevance, including the need to distinguish promising biochemical evidence from established clinical efficacy [33,34]. Although oncology-adjacent prebiotic studies and clinical trial reports are emerging, they remain in the early stages and are context-specific [35,36,37]. Metabolic and microbiome-related evidence includes obese/diabetic animal models, a randomized crossover trial in overweight hypertriglyceridemic adults, and an HIV-associated immune activation protocol [38,39,40]. Antimicrobial activity has been evaluated in vitro [41,42,43], whereas antioxidant and anti-inflammatory outcomes have been supported by focused studies and reviews [44,45,46,47]. Additional preclinical studies have addressed antinociceptive, antiedematogenic, antigenotoxic, antiobesity, and neuroprotective endpoints [48,49,50,51,52], and skin-related exploratory evidence has been reported [53]. These studies have increased interest in camu-camu as a functional food ingredient, but they do not establish disease-treatment claims; health-related interpretations should remain limited to evidence-supported biochemical contexts unless targeted efficacy, bioavailability, dose–response, and intervention data are available.

A comprehensive metabolite inventory of camu-camu is necessary for organizing nutritional and bioactive phytochemicals to develop mechanistic hypotheses that can be tested in future clinical studies. Early phytochemical compilations, including Duke’s records, listed a restricted subset of camu-camu chemicals, whereas more recent phytochemical, food chemistry, processing, and bioaccessibility studies have expanded the documented metabolite list [14,20,54]. Currently, there is no comprehensive metabolite and annotation inventory for camu-camu berries, including the pulp, skin/peel, and seeds. There is a need to integrate literature from previously published records with more complete camu-camu metabolite profiles using complementary analytical chemistry methods.

Previous studies have focused on specific metabolites and bioactive molecules associated with the targeted health outcomes. There is a knowledge gap concerning the comprehensive sampling of metabolites found in camu-camu fruit. Camu-camu has a complex fruit matrix containing organic acids, sugars, amino acids, phenolics, lipids, minerals, volatile constituents, and minor specialized metabolites that cannot be fully represented by a single extraction solvent or analytical platform. Therefore, we used different metabolite extraction methods and analytical approaches to provide a more comprehensive list of camu-camu metabolites. ^1^H NMR provides reproducible structural fingerprints for abundant metabolites and solvent-dependent extraction behavior, whereas LC-MS improves the sensitivity for lower-abundance and structurally diverse compounds. Recent botanical fingerprinting verification studies support the value of combining NMR, LC-MS, and molecular authentication workflows for natural health products and food ingredients [55,56]. The objective of the present study was to assemble an expanded camu-camu metabolite and annotation inventory, including berry pulp, skin/peel, and seed-associated chemistry, by integrating curated literature-derived records with present-study ^1^H NMR and LC-MS observations, and to evaluate solvent-dependent NMR coverage in support of future quality-evaluation and functional-food research.

## 2. Results

### 2.1. Expansion of the Documented Camu-Camu Metabolite and Annotation Inventory

**Figure 1:**
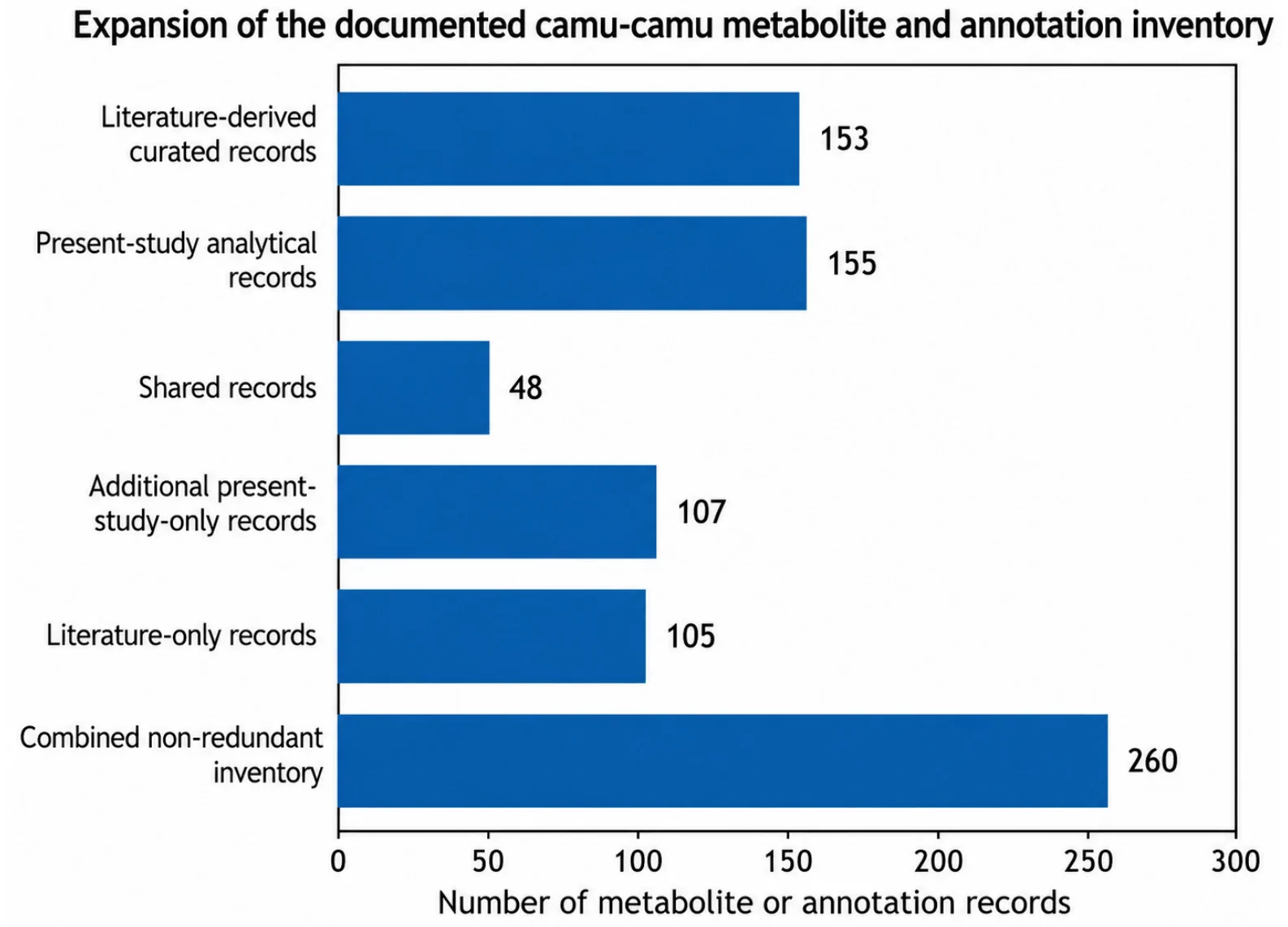
Expansion of the documented camu-camu metabolite and class-level annotations obtained by integrating curated literature-derived records with present-study ^1^H NMR and LC-MS observations. The combined non-redundant inventory contained 260 records.

The curated literature-derived dataset contained 153 non-redundant metabolite or class-level annotation records, whereas the analytical dataset contained 155 records after the removal of one unsupported compound-specific ^1^H NMR assignment. Conservative normalized-name matching identified 48 shared records, 107 additional present-study-only records, and 105 literature-only records. The integration of the two datasets yielded a combined non-redundant inventory of 260 records (Table 1 and Figure 1). These totals include named chemical entities and class-level annotations and should not be interpreted as 260 confirmed compound identities. The revised counts reflect the consolidation of duplicate chemical names, normalization of bibliographic years and analytical methods, and exclusion of literature entries whose cited sources or methods did not support the stated record. This expanded inventory remains consistent with previous camu-camu reviews and composition studies emphasizing vitamin C and organic acids [15,17,21,22,23], phenolics, anthocyanins, ellagitannins, carotenoids, and antioxidant-associated constituents [20,27,28,29,54], as well as selected seed, volatile, and bioactivity-associated compounds [31,32,33,34]. Orthogonal NMR/LC-MS workflows can reveal additional chemical classes [55,56].

### 2.2. Solvent-Dependent ^1^H NMR Annotation Coverage

**Figure 2:**
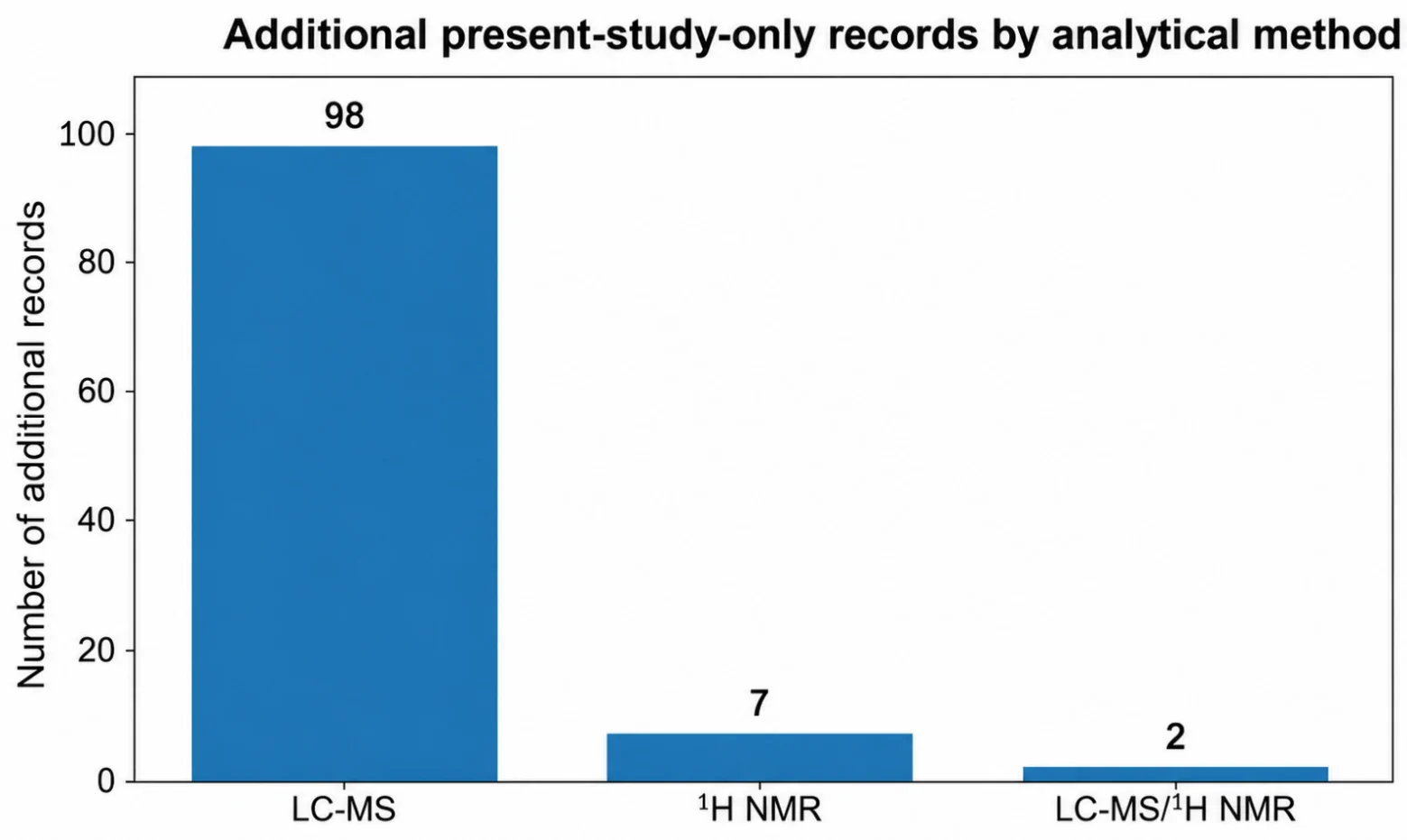
Analytical method distribution of the 107 additional present-study-only records. LC-MS accounted for 98 records, ^1^H NMR for 7 records, and both LC-MS and ^1^H NMR for 2 records. These values are distinct from the solvent-specific ^1^H NMR coverage totals in Table 2 and Tables S2–S9, which count all interpretable annotations per solvent.

Seven solvent systems were evaluated for the extraction and annotation of metabolites using ^1^H NMR spectroscopy (Table 2; Supplementary Figures S1–S7; Tables S2–S9). Each tissue–solvent condition was prepared as three independent extraction replicates (*n* = 3), and the replicate spectra were reviewed for consistent diagnostic regions before the annotations were retained. CH_3_OH:CD_3_OD (9:1, *v*/*v*) provided the broadest annotated coverage, with 28 interpretable metabolite or resonance-class annotations distributed across organic acids, flavanol derivatives, anthocyanins, ellagic- and gallic-acid-related resonances, carbohydrates, amino acids, fatty acid/lipid regions, and other small organic compounds. Acetonitrile-d_3_ yielded 16 assigned records, CD_3_OD:H_2_O (1:1, *v*/*v*) and acetone-d_6_ each yielded 15, H_2_O:D_2_O (1:1, *v*/*v*) yielded 14, DMSO-d_6_ yielded 11, and CDCl_3_ yielded 10 assigned records. These totals represent all interpretable solvent-specific ^1^H NMR annotations, including records already represented in the literature inventory; therefore, they are separate from the analytical method distribution of the 107 additional present-study-only records, as summarized in Figure 2. Methanol-rich extraction has also provided broad botanical metabolite coverage in related studies [55,57].

### 2.3. Chemical Classes and Analytical Methods Represented Among Additional Records

**Figure 3:**
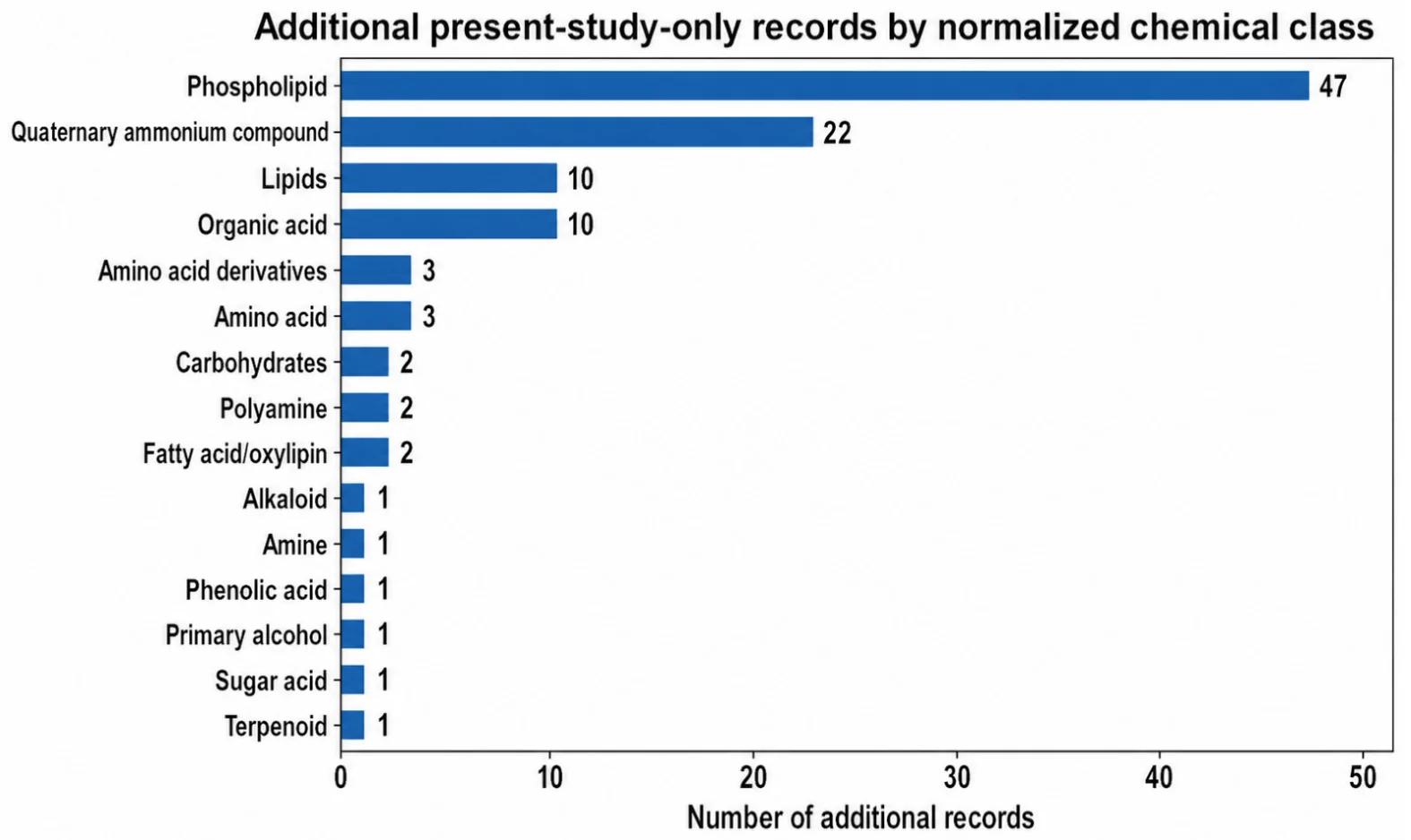
Normalized chemical-class distribution of the newly recorded 107 records relative to the previously curated literature-derived camu-camu inventory. The counts included named compound annotations and explicitly labeled class-level annotations.

The 107 additional present-study-only records were distributed across multiple normalized chemical class categories (Figure 3). Phospholipids represented the largest normalized class (47 records), followed by quaternary ammonium compounds (22), lipids (10), organic acids (10), amino acid derivatives (3), amino acids (3), carbohydrates (2), polyamines (2), fatty acid/oxylipin records (2), and single-record categories comprising alkaloid, amine, phenolic acid, primary alcohol, sugar acid, and terpenoid annotations. For the same 107 records, LC-MS accounted for 98, ^1^H NMR for 7, and both LC-MS and ^1^H NMR for 2 (Figure 2). Across all 155 present-study records, LC-MS evidence was associated with 131 records (117 LC-MS-only and 14 LC-MS/^1^H NMR); this full-inventory count includes both shared and present-study-only records and is an annotation-record count, not a claim that 131 analytes were independently and absolutely quantified. The larger solvent-specific ^1^H NMR totals in Table 2 and Tables S2–S9 include all interpretable annotations per solvent, including previously reported entities. The revised distribution excludes the former compound-specific docosahexaenoic acid assignment because the observed one-dimensional lipid resonances were not structurally specific. The distribution broadens the documented chemistry beyond the phenolic- and vitamin C-centered emphasis of much of the earlier literature [15,17,20] while complementing reports on phenolics, seed and peel constituents, volatiles, and orthogonal botanical fingerprinting [27,28,29,30,31,32,33,34,54,55,56].

The complementary analytical roles of ^1^H NMR and LC-MS were evident in the present-study-only records. LC-MS accounted for the majority of the additional records, whereas ^1^H NMR contributed a smaller number of annotations supported primarily by interpretable diagnostic resonance regions. The LC-MS-dominated expansion was particularly evident for phospholipid-related, quaternary ammonium, lipid, organic-acid, and amino-acid-related records, reflecting the complementary coverage provided by targeted LC-MS/MS for metabolites that may be less readily resolved or detected by ^1^H NMR. In contrast, ^1^H NMR provided direct spectral information on relatively abundant metabolites and solvent-dependent chemical regions. Thus, the two platforms provided complementary rather than interchangeable information for expanding the camu-camu chemical inventory.

### 2.4. Reviewed ^1^H NMR Annotation Status

Seven selected features and resonance classes were emphasized for manuscript-level reporting because their characteristic ^1^H NMR regions were consistently interpretable across the solvent-specific assignment tables and reference-checking workflow (Table 3; Tables S3–S11). These include ethanol, formic acid, β-glucose, γ-aminobutyric acid (GABA), choline, unsaturated fatty acid/lipid resonance classes, and tartaric acid. Assignments were treated conservatively: well-resolved resonances were reported as diagnostic or putative diagnostic annotations, whereas overlapping lipid signals were reported only at the class level and required confirmation by authentic standards, two-dimensional NMR, gas chromatography, or targeted MS/MS when molecular species specificity was required [58,59,60].

## 3. Discussion

The integrated dataset broadens the documented metabolome inventory of camu-camu beyond vitamin C- and phenolic-focused literature. Reviews and composition studies have consistently described camu-camu as a fruit rich in vitamin C, organic acids, and other nutritional phytochemicals [15,17,21,22,23]. Other studies have documented phenolics, anthocyanins, ellagitannins, and carotenoids [20,27,28,29], while seed, peel, and volatile-fraction studies have documented additional specialized constituents [31,32,54]. These constituents remain central to the nutritional and quality narrative; however, the present inventory also contains lipid-related features, quaternary ammonium compounds, amino acid derivatives, organic acids, and other primary metabolism products. Because the inventory intentionally contains both named chemical entities and class-level annotations, its numerical total should not be interpreted as 260 fully structure-confirmed compounds.

The solvent comparison supports the fit-for-purpose extraction. CH_3_OH:CD_3_OD (9:1, *v*/*v*) yielded the broadest NMR coverage in this dataset. Related cross-botanical work has also reported broad NMR/LC-MS coverage for methanol-rich solvent systems, including *Myrciaria dubia* extracts [55], while a separate camu-camu extraction study showed that solvent and tissue influence phenolic recovery [57]. Complementary solvents remain useful for emphasizing particular chemical regions, especially nonpolar lipid signals.

The additional records were not dominated by flavonoids or anthocyanins. Instead, much of the newly represented analytical space comprises phospholipids, quaternary ammonium compounds, amino acid derivatives, organic acids, polyamines, and related lipid/primary metabolism products. This does not replace the established importance of vitamin C and organic acids [17,21,22,23], phenolics, anthocyanins, and ellagitannins [20,27,28,29], or seed/peel constituents [31,32]; rather, it indicates that earlier targeted studies captured an important part of the broader analytical profile of the fruit. For quality control, molecular-species claims should be restricted to records supported by authentic standards or sufficiently diagnostic spectra [55,56].

Several newly recorded metabolite annotations have been reported to be biochemically relevant to brain health-related pathways, providing mechanistic hypotheses for future studies. These annotations include compounds associated with neuronal membrane integrity (choline, phosphatidylcholines, and lysophosphatidylcholines), mitochondrial energy metabolism (carnitine derivatives and β-hydroxybutyrate), neurotransmission (GABA), and cellular stress responses (Table S10) [61,62,63,64,65,66,67,68,69]. When considered together with previously reported camu-camu metabolites, such as quercetin, EGCG, resveratrol, luteolin, naringenin, and ω-3 fatty acid-related metabolites, the expanded inventory encompasses multiple neurobiological pathways that can be evaluated in experimental models. Current clinical evidence supporting the use of camu-camu as a health supplement for treating brain injury is lacking. Therefore, these records should be considered in future targeted studies evaluating the mechanistic hypotheses related to brain health.

The analytical interpretation of these results requires caution. Three independent extraction replicates were prepared for each ^1^H NMR tissue–solvent condition and were used to assess reproducibility of diagnostic resonance regions. The targeted DI/LC-MS/MS analysis comprised samples in different solvents. The analytical plate included one blank, three zero samples, seven calibration standard wells, and three quality control wells. The cited analytical method describes seven calibration levels (Cal 1–Cal 7) and low-, medium-, and high-concentration quality control materials, with method-validation criteria of accuracy within 100 ± 20%, precision CV < 20%, recovery generally within 80–120%, and carry-over assessment after the highest calibrator [70]. Because the study endpoints were annotation presence, annotation coverage, and curated record counts rather than quantitative comparisons among experimental groups, inferential statistical tests were not applied. NMR annotations in complex plant matrices can be affected by resonance overlap, pH, solvent-dependent chemical-shift variation, and co-extracted compounds, while LC-MS annotations can be influenced by isobaric species, adduct formation, matrix effects, and database-matching ambiguity. Therefore, the workflow distinguishes diagnostic annotations from putative or class-level assignments and follows metabolomics reporting resources and databases [58,59,60,71]. Orthogonal agreement was treated as supporting evidence rather than a substitute for pure standards or two-dimensional NMR when isomers or overlapping signals remained possible.

Recent human studies have highlighted the importance of chemically characterizing camu-camu, but they also emphasize the need for caution. A randomized crossover trial in overweight, hypertriglyceridemic adults reported that camu-camu supplementation reduced liver-fat and liver injury markers and altered gut microbiota composition [39]. These findings concern a specific intervention and do not validate every metabolite reported in the present analytical dataset. Therefore, the present manuscript uses such studies only to justify future targeted, quantitative, and bioavailability-focused research, not to claim clinical efficacy from the metabolite and annotation inventory alone.

## 4. Materials and Methods

### 4.1. Sample Preparation and Solvent Extraction

Wild camu-camu (*Myrciaria dubia*) fruit was hand-harvested from riverbank and floodplain-lake habitats in five Brazilian Amazon collection regions: Manaus, Amazonas (approximately 3.1190° S, 60.0217° W); Iranduba, Amazonas (approximately 3.2850° S, 60.1858° W); Manacapuru, Amazonas (approximately 3.3042° S, 60.6175° W); Boa Vista, Roraima (approximately 2.8164° N, 60.6664° W); and the Pesqueiro area near Soure, Pará (approximately 0.7330° S, 48.5170° W). Fruits from the collection regions were pooled and pureed with the seeds at a processing facility in Manaus. The puree was dried using a commercial Refractance Window Dryer (RWD; Tacoma, WA, USA), and the resulting dried shards were milled to pass through a 300-mesh screen and vacuum packed in a metallized Mylar film. The dried berry powder was authenticated as *M. dubia* using orthogonal DNA-based identification and ^1^H NMR metabolite fingerprinting workflows adapted from previously published ingredient-verification methods. No evidence of adulterant species was detected using the applied tests [56,72]. Because fruits from the five collection regions were pooled before processing, the present study did not assess location-specific chemical variations.

The pooled whole-fruit powder was analyzed as an unfractionated preparation. The study also included peel/skin, pulp, and seed preparations derived from authenticated fruit material for tissue-resolved ^1^H NMR analyses. For the NMR solvent comparison, each tissue/preparation and solvent condition was independently extracted and analyzed in triplicate (*n* = 3). For LC-MS, methanol, water, methanol–water (1:1, *v*/*v*), acetone, acetonitrile, and chloroform extracts were prepared, and samples in different solvents were analyzed by targeted quantitative reverse-phase LC-MS/MS followed by direct-flow-injection tandem mass spectrometry. Approximately 300 mg (± 1 mg) of dried material was weighed into microcentrifuge tubes and extracted with 2.0 mL of the designated solvent. The NMR solvent systems were CH_3_OH:CD_3_OD (9:1, *v*/*v*), CD_3_OD:H_2_O (1:1, *v*/*v*), acetone-d_6_, acetonitrile-d_3_, CDCl_3_, DMSO-d_6_, and H_2_O:D_2_O (1:1, *v*/*v*). The samples were vortex-mixed for 30 s, sonicated for 15 min at 25 °C, and centrifuged at 13,000× *g* for 10 min. The clarified supernatants were transferred to clean tubes for NMR or filtered through 0.22 µm PTFE syringe filters for LC-MS. The extraction design followed the botanical-fingerprinting workflow described previously [55].

### 4.2. ^1^H NMR Data Acquisition and Processing

For ^1^H NMR analysis, clarified aliquots were transferred into 5 mm NMR tubes. The deuterated component of each solvent system provided the field-frequency lock: 10% CD_3_OD for CH_3_OH:CD_3_OD (9:1, *v*/*v*), CD_3_OD for CD_3_OD:H_2_O (1:1, *v*/*v*), and the designated deuterated solvent for acetone-d_6_, acetonitrile-d_3_, CDCl_3_, DMSO-d_6_, and H_2_O:D_2_O (1:1, *v*/*v*). Unless otherwise stated, the same acquisition and processing parameters were applied to all the solvent-specific spectra. Spectra were acquired at 400.3 MHz on a Bruker Avance III 400 spectrometer using the one-dimensional NOESY presaturation pulse program noesygpps1d.comp1 under TopSpin 3.6.3 software. The acquisition parameters were as follows: 64 scans (NS = 64), four dummy scans (DS = 4), a 15 s relaxation delay (D1 = 15 s), a spectral width of 20.5437 ppm (8223.684 Hz), 65,536 time-domain points (TD), receiver gain 16.32, and a temperature of approximately 300 K. Spectra were processed in TopSpin 4.0.8 after zero-filling to 131,072 points (SI), using exponential line broadening of 0.3 Hz, Fourier transformation, phase correction, baseline correction, and solvent-specific chemical-shift referencing. Representative spectra are provided in Supplementary Figures S1–S7, and the annotations are provided in Tables S3–S9 [55].

### 4.3. ^1^H NMR Peak Annotation and Confidence Criteria

NMR annotation was based on a conservative comparison of chemical shifts, multiplicity patterns, coupling information where reliable, integration plausibility, complementary resonances, and diagnostic resonance regions against HMDB, published camu-camu NMR/LC-MS literature, and metabolomics reporting recommendations [27,28,55,58,59]. Diagnostic regions were checked for reproducibility across the triplicate extracts. Compound-level assignments were retained when supported by characteristic NMR features and/or concordant LC-MS evidence from matched aliquots of the same authenticated sample preparations. LC-MS support included retention-time and MRM-transition agreement with authentic standards where available. Orthogonal agreement between NMR and LC-MS was treated as increased support but not as a replacement for a pure standard or two-dimensional NMR, where isomers or overlapping resonances remained possible. Table S11 separates diagnostic or putative diagnostic annotations from class-level lipid region annotations and identifies records that require additional targeted confirmation.

### 4.4. Targeted LC-MS/MS Analysis

Targeted LC-MS/MS was performed on samples in different solvents using reverse-phase LC-MS/MS followed by direct-flow-injection tandem mass spectrometry [70]. The analytical panel screened 209 metabolites. The analysis was performed using an AB Sciex 4000 QTRAP (Foster City, CA, USA) coupled to an Agilent 1260 UHPLC (Agilent Technologies, Palo Alto, CA, USA), multiple-reaction-monitoring detection, calibration standards, isotope-labeled and other internal standards, a 96-well plate format, and the Analyst 1.6.2 software. The analytical plate contained one blank, three zero samples, seven calibration standard wells, and three quality control wells. The final inventory contains 131 records with LC-MS evidence (117 LC-MS-only and 14 supported by both LC-MS and ^1^H NMR). The quantitative measurements were used to support metabolite and annotation coverage; concentration values are not tabulated in this article.

The analytical method (Supplementary Methods S1–S3) used phenyl isothiocyanate derivatization for primary- and secondary-amine-containing metabolites and 3-nitrophenylhydrazine derivatization for keto- and carboxyl-containing metabolites [70]. For the phenyl isothiocyanate branch, 20 µL of internal-standard mixture and 10 µL of sample extract were dried under nitrogen for 30 min, reacted with 50 µL of 5% phenyl isothiocyanate for 20 min at room temperature, dried for 1 h, extracted with 300 µL of 5 mM ammonium acetate in methanol, shaken at 450 rpm for 30 min, and centrifuged at 50× *g* for 5 min. For LC-MS/MS, 150 µL of extract was diluted with 150 µL of water; the remaining extract was diluted with 400 µL of direct-flow-injection buffer for acylcarnitine and C-6-sugar analysis. Phospholipid preparation used 10 µL of internal-standard mixture, 5 µL of plant extract, 150 µL of extraction solution, and 600 µL of direct-flow-injection buffer. For organic acids, 10 µL of internal-standard mixture, 10 µL of sample extract, and 30 µL of 75% aqueous methanol were combined with 25 µL each of 250 mM 3-nitrophenylhydrazine, 150 mM EDC, and 7.5% pyridine, shaken at 450 rpm for 2 h at room temperature, and diluted with 350 µL of water and 25 µL of BHT solution (2 mg/mL in methanol).

Amino acids and biogenic amines were separated on an Agilent Zorbax Eclipse XDB C18 column Palo Alto, CA, USA (3.0 × 100 mm, 3.5 µm, 80 Å) with a C18 guard column using 0.2% formic acid in water and acetonitrile, a 0.5 mL/min flow rate, 10 µL injection, 50 °C column temperature, positive-electrospray scheduled MRM, +5500 V IonSpray voltage, 500 °C source temperature, CUR/GAS1/GAS2 settings of 20/40/50, CAD medium, and EP +15 V. Organic acids used the same analytical column, 0.01% formic acid in water and acetonitrile, a 0.3 mL/min flow rate, 10 µL injection, 40 °C, negative-electrospray scheduled MRM, −4500 V, 400 °C, CUR/GAS1/GAS2 settings of 20/30/30, CAD medium, and EP −10 V. Direct-flow-injection MS/MS used 20 µL injections, positive MRM for phospholipids and acylcarnitines, negative MRM for C-6 sugars, +5500/−4500 V, a 200 °C source temperature, CUR/GAS1/GAS2 settings of 20/40/50, CAD medium, and EP/CXP values of +10/+15 V in positive mode and −10/−15 V in negative mode [70].

The cited analytical method used seven calibration levels (Cal 1–Cal 7) prepared in water or 75% aqueous methanol and corresponding isotope-labeled internal standards for analyte-to-internal-standard peak area ratio quantification [70]. Chemical and isotope-labeled standards were obtained from C/D/N Isotopes Inc. (Pointe-Claire, QC, Canada), Cayman Chemical (Ann Arbor, MI, USA), Toronto Research Chemicals Inc. (Toronto, ON, Canada), Cambridge Isotope Laboratories Inc. (Tewksbury, MA, USA), Sigma-Aldrich Canada Ltd. (Oakville, ON, Canada), and IsoSciences LLC (Ambler, PA, USA); 3-(3-hydroxyphenyl)-3-hydroxypropionic acid (HPHPA) and its ^13^C-labeled analog were synthesized in-house [70]. Amino acids, amino acid derivatives, biogenic amines, and organic acids were absolutely quantified using analyte-specific seven-point calibration curves with quadratic regression and 1/x weighting. Acylcarnitines, C-6 sugars, and phospholipids without individual chemical standards were analyzed semi-quantitatively using representative single-point calibration with linear regression through zero. The cited method used low-, medium-, and high-concentration quality control materials prepared in triplicate, with method-validation criteria of accuracy within 100 ± 20%, precision CV < 20%, recovery generally within 80–120%, and carry-over assessment after the highest calibrator [70]. Analyte-specific Q1/Q3 precursor/product-ion transitions and associated declustering potential (DP), collision energy (CE), and collision-cell exit potential (CXP) settings were used in the targeted method [70]. The numerical project-specific Q1/Q3, DP, CE, and CXP values for the 209-metabolite analytical panel were not included in the analytical records supplied to the authors by the external analytical service; therefore, they were not reported as project-specific values. The published method cited in [70] provides numerical transition parameters for its published assay panel; however, these values were not substituted for the project-specific acquisition table. Supplementary Methods S3 and Table S12 provide the available preparation, chromatographic, source, calibration, standards, quantification, quality control, and transition-reporting details.

### 4.5. Literature-Inventory Curation and Dataset Integration

The literature-derived table and present-study analytical table were curated using conservative name normalization. Exact duplicates and clear orthographic variants were consolidated, and unsupported or incorrectly sourced literature entries were excluded from the curated counts during the working review. Exact normalized name matches were counted as shared records, and high-similarity names were not merged unless they clearly represented the same chemical entity. The resulting counts distinguished literature-only, present-study-only, shared, and combined non-redundant records. The named chemical entities and class-level annotations are listed in Table S1 [73,74,75,76,77,78,79,80,81,82,83,84,85,86] (workbook). HMDB, metabolomics reporting standards, METLIN, and MetaboAnalyst were used to support annotation and data interpretation [58,59,60,71]. Camu-camu-specific anthocyanin and phenolic assignments were further verified by comparing them with published HPLC-PDA/HPLC-MS/NMR and HPLC-MS/MS studies [87,88].

### 4.6. Replication and Statistical Treatment

Three independent extraction replicates were used for each ^1^H NMR tissue–solvent condition to assess reproducibility of diagnostic resonance regions and annotation presence. The LC-MS analysis comprised samples in different solvents analyzed by targeted quantitative reverse-phase LC-MS/MS followed by direct-flow-injection tandem mass spectrometry. Because the study endpoints were a curated metabolite/annotation inventory and descriptive solvent-dependent annotation coverage rather than quantitative comparisons of metabolite concentrations among biological groups, inferential statistical testing was not considered applicable.

## 5. Conclusions

This study assembled a comprehensive camu-camu metabolite and annotation inventory by integrating curated literature evidence with ^1^H NMR and LC-MS observations. The detailed metabolomic record provides a basis for species-specific quality control fingerprints that may support the authentication of camu-camu products in foods and natural health products. The metabolomic library also provides candidate metabolites for future mechanistic studies on the reported health-related effects. The principal conclusions are analytical: solvent choice influences observable coverage, integrated NMR/LC-MS workflows expand documentation, and compound-level confidence must be separated from class-level annotations. Functional-food and neurological interpretations remain hypothesis-generating until targeted quantification, authentic-standard confirmation, bioavailability assessment, and intervention studies are completed. This study expands our understanding of the complex metabolome of camu-camu and provides testable mechanistic hypotheses for future studies.

## Figures and Tables

**Table 1:** Summary of literature-derived and present study record coverage after curation.

Dataset	Number of Records
Literature-derived curated records	153
Present-study analytical records	155
Shared records	48
Additional present-study-only records	107
Literature-only records	105
Combined non-redundant inventory	260

**Table 2:** Solvent systems used for ^1^H NMR-based annotation coverage in camu-camu extracts.

Solvent System	Number of Annotated Records	Major Chemical Groups Represented
CH_3_OH:CD_3_OD (9:1, *v*/*v*)	28	Flavanol derivatives; anthocyanins; ellagic acid; gallic acid; organic acids; fatty acids; amino acids; carbohydrates; other organic compounds
CD_3_OD:H_2_O (1:1, *v*/*v*)	15	Flavanol derivatives; ellagic acid; gallic acid; organic acids; amino acids; carbohydrates; other organic compounds
acetone-d_6_	15	Flavanol derivatives; ellagic acid; gallic acid; organic acids; amino acids; carbohydrates; other organic compounds
acetonitrile-d_3_	16	Flavanol derivatives; ellagic acid; gallic acid; organic acids; amino acids; fatty acids; carbohydrates; other organic compounds
CDCl_3_	10	Lipids and fatty acids
DMSO-d_6_	11	Ellagic acid; organic acids; amino acids; carbohydrates; other organic compounds
H_2_O:D_2_O (1:1, *v*/*v*)	14	Flavanol derivatives; ellagic acid; gallic acid; organic acids; amino acids; carbohydrates; other organic compounds

**Table 3:** Selected diagnostic ^1^H NMR features and reviewed annotation status of camu-camu extracts.

Annotation	Characteristic ^1^H NMR Chemical Shift (δ, ppm)	Annotation Note
Ethanol	1.06–1.20 (t, CH_3_; solvent-dependent)	Putative diagnostic methyl resonance; values reported across Tables S4–S9
Formic acid	8.45–8.46 (s)	Formyl proton
β-Glucose	4.44–4.65 (d, H-1 β-anomer; solvent-dependent)	Anomeric proton region
GABA	2.28 (t, J = 7.4 Hz); 2.45 (t, J = 7.1 Hz)	Methylene resonances; overlap possible
Choline	3.19–3.27 (s, N(CH_3_)_3_; solvent-dependent)	Trimethylammonium resonance
Unsaturated fatty-acid/lipid resonances	0.75–1.01, 1.25–1.30, 1.59–1.69, 1.99–2.04, 2.29–2.33, 2.75–2.84, 4.29, 5.25–5.36	Class-level lipid-region pattern; not compound-specific
Tartaric acid	4.30 (d, J = 2.8 Hz)	Oxygenated methine resonance

## Data Availability

Representative NMR spectra and solvent-specific annotation details are provided in Supplementary Figures S1–S7 and Tables S3–S11. The complete NMR spectral dataset and associated processing files are available from the corresponding author upon reasonable request. Curated metabolite tables and LC-MS annotation summaries are provided in the Supplementary Materials.

## Supplementary Materials

The following supporting information can be downloaded at: https://www.mdpi.com/article/10.3390/molecules31183167/s1, Supplementary Methods S1, sample design, solvent-specific ^1^H NMR acquisition parameters, lock solvents, and annotation notes; Supplementary Methods S2, reference-checking workflow; Supplementary Methods S3, targeted LC-MS/MS preparation, derivatization, acquisition, annotation, quality control, and transition-reporting procedures; Figures S1–S7, solvent-specific annotated ^1^H NMR spectra; Table S1, curated literature-derived metabolite and class-level annotation records; Table S2, solvent systems and annotated ^1^H NMR coverage; Tables S3–S9, solvent-specific ^1^H NMR chemical-shift features and compound/class annotations; Table S10, conservative biochemical context and evidence map for selected annotations; Table S11, reference-checked NMR annotation-status table; Table S12, LC-MS/MS source conditions, MRM acquisition parameters, and project-specific transition-parameter availability. Tables S1–S12 and supporting curation/count worksheets are provided in the accompanying 18-worksheet Excel workbook.
