# Supplementary material for: Assembling an Expanded Metabolome for Camu-Camu (Myrciaria Dubia): Bioactive and Nutritional Compounds Relevant to Functional Food Research
Source: Molecules. 2026 Sep 9;31(18):3167. doi: 10.3390/molecules31183167 (PMC13609166; doi:10.3390/molecules31183167)
Supplement: Supplementary file 1 [file molecules-31-03167-s001.zip › molecules-4469110-supplementary.pdf]

Supplementary Materials

Assembling an Expanded Metabolome for Camu-Camu (*Myrciaria dubia*): Bioactive and Nutritional Compounds Relevant to Functional Food Research

Subramanyam Ragupathy [1], Varathan Vinayagam [1], Velautham Saravanan [1], Arunachalam Thirugnanasambandam [1,2] and Steven G. Newmaster [1,*]

[1] College of Biological Science, University of Guelph, Guelph, Ontario, Canada, N1G 2W1; ragu@uoguelph.ca(S.R.); vinaychemist89@gmail.com (V.V.); saravananorganic2014@gmail.com(V.S.); athiru@outlook.com(A.T)
[2] Biological and Life Sciences, Canadian Light Source, Saskatoon, Saskatchewan, Canada, S7N 2V3
* Correspondence: snewmast@uoguelph.ca

This file contains sample and replicate information, exact solvent-specific ^{1}H NMR acquisition and processing metadata, a reference-checking workflow, an expanded targeted LC-MS/MS and quality-control method, individual solvent-specific spectra, figure/table pairings, and supporting tables. Tables S1-S11 and supporting curation/count worksheets are provided in the accompanying 18-worksheet Excel workbook. Table S12 is included in this document. References are numbered independently and include only the sources cited in the supplementary text and tables.

Supplementary Methods S1. Solvent-specific ^{1}H NMR annotation notes

Wild camu-camu (*Myrciaria dubia*) fruit was hand-harvested from riverbank and floodplain-lake habitats in five Brazilian Amazon collection regions: Manaus, Amazonas (approximately 3.1190° S, 60.0217° W); Iranduba, Amazonas (approximately 3.2850° S, 60.1858° W); Manacapuru, Amazonas (approximately 3.3042° S, 60.6175° W); Boa Vista, Roraima (approximately 2.8164° N, 60.6664° W); and the Pesqueiro area near Soure, Pará (approximately 0.7330° S, 48.5170° W). Fruits from the collection regions were pooled and pureed with the seeds at a processing facility in Manaus. The puree was dried using a commercial Refractance Window Dryer (RWD), and the resulting dried shards were milled to pass a 300-mesh screen and vacuum-packed in metallized Mylar film. The dried berry powder was authenticated as *M. dubia* using orthogonal DNA-based identification and ^{1}H NMR metabolite fingerprinting workflows adapted from previously published ingredient-verification methods. No evidence of adulterant species was detected using the applied tests [56,72]. Because fruits from the five collection regions were pooled before processing, this study did not evaluate locality-specific chemical variations.

The pooled whole-fruit powder was analyzed as an unfractionated preparation, and the study also included peel/skin, pulp, and seed preparations derived from authenticated fruit material. For the ^{1}H NMR solvent comparison, each tissue–solvent condition was independently extracted and analyzed in triplicates (n = 3). Triplicate spectra were used to assess the reproducibility of the diagnostic resonance regions and annotation presence. LC-MS analysis was performed on samples in different solvents using targeted quantitative reverse-phase LC-MS/MS followed by direct-flow-injection tandem mass spectrometry. Inferential statistical testing was not applied because the endpoints were annotation presence and solvent-dependent annotation coverage rather than quantitative comparisons among biological groups. Solvent-dependent ^{1}H NMR annotations were interpreted

using diagnostic resonance positions, multiplicity patterns where reliable, and coupling information where sufficiently resolved. $CH_3OH:CD_3OD$ (9:1, v/v) provided the broadest coverage. Polar solvent systems emphasized organic acids, carbohydrates, amino acids, and phenolic regions, whereas $CDCl_3$ emphasized lipid and fatty-acyl-chain resonances. Compound-specific assignments were avoided when resonance overlap or solvent/matrix-dependent shifts prevented unique structural identification.

All solvent-specific spectra were acquired using the same instrument and core parameters, unless otherwise noted. Spectra were collected at 400.3 MHz on a Bruker Avance III 400 spectrometer using noesygpps1d.comp1 under TopSpin 3.6.3, with NS = 64, DS = 4, D1 = 15 s, a spectral width of 20.5437 ppm (8223.684 Hz)), TD = 65,536, receiver gain = 16.32, and temperature of approximately 300 K. Processing in TopSpin 4.0.8 used SI = 131,072 and exponential line broadening of 0.3 Hz before Fourier transformation, phase correction, baseline correction, and solvent-specific referencing. The deuterated lock component was 10% CD_3OD in $CH_3OH:CD_3OD$ (9:1, v/v), CD_3OD in $CD_3OD:H_2O$ (1:1, v/v), acetone-d_6, acetonitrile-d_3, $CDCl_3$, DMSO-d_6, and D_2O in $H_2O:D_2O$ (1:1, v/v).

The solvent-specific ^{1}H NMR counts in Table S2 and Tables S3-S9 represent all interpretable annotations per solvent, including records already represented in the curated literature-derived inventory. They are not analytical method counts for the 107 additional present-study-only records summarized in main-manuscript Figure 3.

Supplementary Methods S2. Reference-checking workflow for ^{1}H NMR peak annotations

Peak annotations were reviewed against HMDB, published reference spectra, camu-camu NMR/LC-MS literature, and metabolomics reporting recommendations [55,58,59,87,88]. The diagnostic regions were checked for reproducibility across triplicate NMR preparations. LC-MS support used analyte-specific MRM transitions and the calibration/internal standard procedures described in [70]. Records observed by both ^{1}H NMR and LC-MS were treated as orthogonally supported annotations. The diagnostic and class-level resonances are summarized in Table S11, and detailed solvent-specific annotations are provided in Tables S3-S9.

Supplementary Methods S3. Targeted LC-MS/MS assay and quality control

Targeted quantitative metabolomics was performed on samples in different solvents using reverse-phase LC-MS/MS, followed by direct-flow injection tandem mass spectrometry [70]. The analytical panel screened 209 metabolites in the samples. The analysis used an AB Sciex 4000 QTRAP coupled to an Agilent 1260 UHPLC, multiple-reaction-monitoring detection, calibration standards, isotope-labeled and other internal standards, a 96-well plate format, and the Analyst 1.6.2 software. The analytical plate contained one blank, three zero samples, seven calibration standard wells, and three quality control wells. The final inventory contained 131 records with LC-MS evidence (117 LC-MS-only and 14 supported by both LC-MS and ^{1}H NMR), and quantitative measurements were used to support metabolite and annotation coverage.

Chemicals and standards. LC/MS-grade formic acid, methanol, acetonitrile, water, and ammonium acetate were used for chromatographic separation and extraction of the samples. Ethanol, pyridine, phenyl isothiocyanate (PITC), 3-nitrophenylhydrazine (3-NPH), 1-ethyl-3-(3-dimethylaminopropyl)carbodiimide (EDC), and butylated hydroxytoluene (BHT) were used for the derivatization and stabilization of the samples. In the cited analytical method, individual chemical standards and corresponding isotope-labeled internal standards were obtained from C/D/N Isotopes Inc., Cayman Chemical, Toronto Research Chemicals, Cambridge Isotope Laboratories Inc., Sigma-Aldrich, and

IsoSciences; stock solutions and internal-standard working mixtures were prepared at analyte-specific concentrations; and Cal 1–Cal 7 were prepared in water or 75% aqueous methanol, aliquoted, and stored at −86 °C [70].

Two in-plate derivatization procedures were performed. For the phenyl isothiocyanate branch, 20 µL of internal-standard mixture and 10 µL of sample extract were dried under nitrogen for 30 min, reacted with 50 µL of 5% phenyl isothiocyanate for 20 min at room temperature, dried for 1 h, extracted with 300 µL of 5 mM ammonium acetate in methanol, shaken at 450 rpm for 30 min, and centrifuged at 50 × g for 5 min. For LC-MS/MS, 150 µL of the extract was diluted with 150 µL of water. The remaining extract was diluted with 400 µL of direct-flow injection buffer for acylcarnitine and C-6-sugar analysis. For phospholipids, 10 µL of the internal standard mixture and 5 µL of the plant extract were combined with 150 µL of the extraction solution and 600 µL of the direct-flow injection buffer. For organic acids, 10 µL of internal-standard mixture, 10 µL of sample extract, and 30 µL of 75% aqueous methanol were combined with 25 µL each of 250 mM 3-nitrophenylhydrazine, 150 mM EDC, and 7.5% pyridine, shaken at 450 rpm for 2 h at room temperature, and diluted with 350 µL of water and 25 µL of BHT solution (2 mg/mL in methanol) [70].

Amino acids and biogenic amines were separated on an Agilent Zorbax Eclipse XDB C18 column (3.0 × 100 mm, 3.5 µm, 80 Å) with a C18 guard column. The mobile phases were 0.2% formic acid in water (A) and 0.2% formic acid in acetonitrile (B); the gradient was 100% A at 0–0.5 min, 5% A at 5.5–6.5 min, and 100% A at 7.0–9.5 min. The flow rate was 0.5 mL/min, injection volume was 10 µL, and column temperature was 50 °C. Positive electrospray scheduled MRM was performed using +5500 V, 500 °C, CUR/GAS1/GAS2 of 20/40/50, CAD medium, and EP +15 V. Organic acids were analyzed using 0.01% formic acid in water (A) and acetonitrile (B); the gradient was 70% A at 0–1.5 min, 5% A at 8.0 min, 0% A at 8.01–9.5 min, and 70% A at 9.6–14.0 min. The flow rate was 0.3 mL/min, injection volume was 10 µL, and column temperature was 40 °C. Negative electrospray scheduled MRM used −4500 V, 400 °C, CUR/GAS1/GAS2 of 20/30/30, CAD medium, and EP −10 V [70].

Direct-flow-injection MS/MS used a mobile phase composed of 60 µL formic acid, 10 mL water, and 290 mL methanol, with a flow program of 0.03 mL/min at 0–1.6 min, 0.2 mL/min at 2.4–2.8 min, and 0.03 mL/min at 3.0 min. The injection volume was 20 µL. Positive MRM was used for phospholipids and acylcarnitines, and negative MRM was used for C-6 sugars. The positive mode used +5500 V, 200 °C, CUR/GAS1/GAS2 of 20/40/50, CAD medium, EP +10 V, and CXP +15 V; the negative mode used −4500 V, 200 °C, the same gas settings, EP −10 V, and CXP −15 V. Analyte-specific Q1/Q3 precursor/production pairs and associated declustering potential (DP), collision energy (CE), and collision cell exit potential (CXP) settings were optimized for the cited analytical method [70]. The numerical project-specific Q1/Q3, DP, CE, and CXP values for the 209-metabolite analytical panel were not included in the analytical records supplied to the authors by the external analytical service and are therefore not reproduced as project-specific values. The published method [70] provides numerical transition parameters for its published assay panel; these values were not substituted for the project-specific acquisition table.

Amino acids, amino-acid derivatives, biogenic amines, and organic acids were quantified using analyte-specific seven-point calibration curves and corresponding isotope-labeled internal standards, with quadratic regression and 1/x weighting. Acylcarnitines, C-6 sugars, and phospholipids without individual chemical standards were analyzed semi-quantitatively using representative single-point calibration with linear regression through zero. The cited method used low-, medium-, and high-concentration quality control materials prepared in triplicate. The method-validation acceptance criteria were accuracy within 100 ± 20% and precision CV < 20%; recovery was generally 80–120%, and carry-

over was assessed by comparing the highest calibration-level injection with the following double blank [70]. The analytical conditions, standards, calibration procedures, quality control parameters, and availability of project-specific transition parameters are summarized in Table S12.

Table S1. Curated literature-derived camu-camu metabolite and class-level annotation records.

Metabolite/annotation	Chemical class	Plant Tissue	Analytical method	Published references
**(E)-α-Bergamotene**	Sesquiterpene	Fruit	HS-SPME-GC/MS	Souza et al. (2021)
**(E)-β-Farnesene**	Sesquiterpene	Fruit	HS-SPME-GC/MS	Souza et al. (2021)
**(Iso)liquiritigenin-O-hexoside**	Flavonoid	Fruit	LC-MS/MS	Conceição et al. (2020)
**α-Glucose**	Monosaccharide	Fruit	Carbohydrate/compositional analysis	Zapata and Dufour (1993); Akter et al. (2011)
**1-Methylmalate**	Organic acid ester	Fruit juice	Bioactivity-guided identification	Castro et al. (2020)
**2,4-Dihydroxybenzoic acid**	Phenolic acid	Seed coat	HPLC	Fidelis et al. (2018); Conceição et al. (2020)
**2,5-Dihydroxybenzoic acid**	Phenolic acid	Seed coat	HPLC	Fidelis et al. (2018)
**3-Carene**	Monoterpene	Fruit	GC-MS	Quijano and Pino (2007)
**4-Aminobutanoate**	Amino acid	Fruit	HPLC-PDA; HPLC-PDA-MS	Zapata and Dufour (1993)
**4-O-Caffeoylquinic acid**	Hydroxycinnamic acid derivative	Peel	LC-MS/MS	Conceição et al. (2020)
**4-O-methylellagic acid**	Polyphenol	Fruit	HPLC-DAD	Chirinos et al. (2010)
**5-O-caffeoylquinic acid**	Phenolic compound	Fruit	LC-MS/MS	Conceição et al. (2020)
**Acetic acid**	Organic acid	Fruit	HPLC-DAD	Chirinos et al. (2010)
**Aconitic acid**	Organic acid	Fruit	HPLC-DAD	Chirinos et al. (2010)
**Acylphloroglucinols**	Phloroglucinol	Fruit	GC-MS; HPLC	Kaneshima et al. (2017)
**Alanine**	Amino acid	Pulp	Amino-acid composition analysis; review-derived record	Zapata and Dufour (1993); Akter et al. (2011)
**Alnusiin**	Ellagitannin	Extract	UHPLC-QTOF-MS/MS	Rodrigues et al. (2020)
**Anthocyanins**	Flavonoid	Fruit	LC-MS/MS	Conceição et al. (2020)
**Apigenin-O-hexoside**	Flavonoid	Fruit	LC-MS/MS	Conceição et al. (2020)
**Arginine**	Amino acid	Pulp	Amino-acid composition analysis; review-derived record	Zapata and Dufour (1993); Akter et al. (2011)
**Ascorbic acid**	Vitamin C	Pulp, peel	HPLC-DAD/PDA; compositional analysis	Justi et al. (2000); Zapata and Dufour (1993); Fracassetti et al. (2013); Chirinos et al. (2010)
**Asparagine**	Amino acid	Fruit	Amino-acid composition analysis; review-derived record	Zapata and Dufour (1993); Akter et al. (2011)
**Aspartic acid**	Amino acid	Pulp	Amino-acid composition analysis; review-derived record	Zapata and Dufour (1993); Akter et al. (2011)
**Benzoic acid**	Organic acid	Fruit	LC-MS/MS	Conceição et al. (2020)
**Butyric acid**	Organic acid	Fruit	LC-MS/MS	Conceição et al. (2020)
**Caffeic acid**	Phenolic acid	Seed coat	HPLC	Fidelis et al. (2018); Conceição et al. (2020)
**Camphene**	Monoterpene	Fruit	GC-MS	Quijano and Pino (2007)
**Castalagin**	Ellagitannin	Seed	HPLC-DAD-ESI-MS/MS	Fracassetti et al. (2013); Conceição et al. (2020); Fujita et al. (2015); Fidelis et al. (2020)

**Casuarictin**	Ellagitannin	Seed	HPLC-DAD-ESI-MS/MS; UPLC-HR-QTOF-MS/MS	Fracassetti et al. (2013)
**Catechin**	Flavan-3-ol	Fruit, seed	HPLC-DAD-ESI-MS/MS; HPLC-DAD	Fracassetti et al. (2013); Chirinos et al. (2010)
**Chlorogenic acid**	Phenolic acid	Seed coat	HPLC	Fidelis et al. (2018); Rodrigues et al. (2020)
**Citric acid**	Organic acid	Fruit	Organic acid analysis (HPLC)	Akter et al. (2011); Langley et al. (2015); Zapata and Dufour (1993); Nascimento et al. (2013)
**Cyanidin derivatives**	Anthocyanidin	Fruit	LC-TOF-MS; LC-MS/MS	Fujita et al. (2015); Conceição et al. (2020)
**Cyanidin-3-O-glucoside**	Anthocyanin	Pulp, peel; Fruit	HPLC-DAD-MS; LC-MS; Amino acid analyzer	Zanatta et al. (2005); Chirinos et al. (2010); Conceição et al. (2020)
**d-Limonene**	Monoterpene	Fruit	HS-SPME-GC/MS	Souza et al. (2021); Conceição et al. (2020)
**Dehydroascorbic acid**	Vitamin C metabolite	Pulp; Fruit	HPLC-DAD; compositional analysis; HPLC-PDA; HPLC-PDA-MS	Fracassetti et al. (2013); Zapata and Dufour (1993)
**Delphinidin-3-O-glucoside**	Anthocyanin	Pulp, peel; Fruit	HPLC-DAD-MS; LC-MS; Amino acid analyzer	Zanatta et al. (2005); Chirinos et al. (2010); Conceição et al. (2020)
**Di-HHDP-galloyl-glucose (casuarictin/potentillin)**	Ellagitannin	Fruit	LC-MS/MS	Conceição et al. (2020)
**Di-HHDP-galloyl-glucoside**	Polyphenol	Fruit	LC-MS/MS	Conceição et al. (2020)
**Ellagic acid**	Phenolic acid	Pulp, peel, seed	HPLC-DAD-ESI-MS/MS; UPLC-HR-QTOF-MS/MS; HPLC-DAD	Fracassetti et al. (2013); Chirinos et al. (2010); Conceição et al. (2020); Fujita et al. (2015); Akter et al. (2011); Rodrigues et al. (2020); Fidelis et al. (2020)
**Ellagic acid hexoside**	Phenolic glycoside	Seed	HPLC-DAD-ESI-MS/MS; LC-MS/MS	Fracassetti et al. (2013); Conceição et al. (2020)
**Ellagic acid pentoside**	Phenolic glycoside	Seed	HPLC-DAD-ESI-MS/MS; LC-MS/MS	Fracassetti et al. (2013); Conceição et al. (2020)
**Epicatechin**	Flavan-3-ol	Fruit, seed	HPLC-DAD-ESI-MS/MS	Fracassetti et al. (2013)
**Epicatechin-3-gallate**	Flavan-3-ol gallate	Peel; seed	NMR; degradation/thiolysis	Kaneshima et al. (2017)
**Epigallocatechin-3-gallate**	Flavan-3-ol gallate	Peel; seed; Fruit	NMR; degradation/thiolysis; HPLC; UHPLC-QTOF-MS/MS	Kaneshima et al. (2017); Rodrigues et al. (2020)
**Ethyl butyrate**	Organic Molecules	Fruit	HS-SPME-GC/MS	Souza et al. (2021)
**Eucalyptol (1,8-cineole)**	Monoterpene oxide	Fruit	Database record; GC-MS literature context	Duke phytochemical database
**Fatty acid (Oleic acid, Linoleic acid, Linolenic acid, Saturated fatty acids)**	Fatty acids	Fruit	1H-NMR, 13C-NMR	Filho et al. (2018)
**Ferulic acid**	Phenolic compound	Fruit	LC-MS/MS	Conceição et al. (2020)
**Ferulic acid hexoside**	Hydroxycinnamate conjugate	Pulp	LC-MS/MS	Conceição et al. (2020)
**Flavanols derivatives**	Flavonoid	Fruit	LC-MS/MS	Conceição et al. (2020)
**Fructose**	Sugar	Fruit	Carbohydrate analysis	Akter et al. (2011); Langley et al. (2015); Zapata and Dufour (1993)
**Fumaric acid**	Organic acid	Fruit	HPLC-DAD	Chirinos et al. (2010)
**Gallic acid**	Phenolic acid	Fruit	HPLC-DAD-ESI-MS/MS; HPLC-DAD	Fracassetti et al. (2013); Chirinos et al. (2010); Fujita

				et al. (2015); Rodrigues et al. (2020); Fidelis et al. (2020)
**Gallocatechin**	Polyphenols	Fruit	UHPLC-QTOF-MS/MS	Rodrigues et al. (2020)
**Glucose**	Sugar	Fruit	Carbohydrate analysis	Akter et al. (2011); Langley et al. (2015); Zapata and Dufour (1993)
**Glutamate**	Amino acid	Fruit	HPLC-PDA; HPLC-PDA-MS	Zapata and Dufour (1993); Akter et al. (2011)
**Glutamic acid**	Amino acid	Pulp	Amino-acid composition analysis; review-derived record	Zapata and Dufour (1993); Akter et al. (2011)
**Glutamine**	Amino acid	Fruit	Review-derived amino-acid composition record	Akter et al. (2011)
**Glycine**	Amino acid	Fruit	Amino-acid composition analysis; review-derived record	Zapata and Dufour (1993); Akter et al. (2011)
**Humulene**	Terpene	Fruit	GC-MS	Quijano and Pino (2007)
**Isocitric acid**	Organic acid	Fruit	Organic acid analysis (HPLC)	Akter et al. (2011); Langley et al. (2015); Zapata and Dufour (1993)
**Isoleucine**	Amino acid	Fruit	Amino-acid composition analysis; review-derived record	Zapata and Dufour (1993); Akter et al. (2011)
**Isomyrtucommulone B**	Acylphloroglucinol	Peel; seed	Isolation; NMR	Kaneshima et al. (2017); Castro et al. (2020)
**Leucine**	Amino acid	Pulp	Amino-acid composition analysis; review-derived record	Akter et al. (2011); Zapata and Dufour (1993)
**Linoleic acid**	Fatty acid (ω-6)	Seed oil; Fruit	GC-FID; ^{1}H/^{13}C NMR; 1H-NMR, 13C-NMR	Justi et al. (2000); Filho et al. (2018)
**Lutein**	Carotenoid	Peel	HPLC-PDA; HPLC-PDA-MS	Zanatta and Mercadante (2007)
**Luteolin**	Flavone	Pulp; peel; seed	UPLC-ESI-MS/MS	Cunha-Santos et al. (2022); Conceição et al. (2020)
**Luteoxanthin**	Carotenoid	Peel	HPLC-PDA; HPLC-PDA-MS	Zanatta and Mercadante (2007)
**Lycopene**	Carotenoid	Peel	HPLC-PDA; HPLC-PDA-MS	Zanatta and Mercadante (2007)
**Lysine**	Amino acid	Fruit	Amino-acid composition analysis; review-derived record	Zapata and Dufour (1993); Akter et al. (2011)
**Magnesium (Mg)**	Mineral	Seed	ICP-OES	Filho et al. (2018); Freitas et al. (2019)
**Malic acid**	Organic acid	Fruit	Organic acid analysis (HPLC)	Akter et al. (2011); Langley et al. (2015); Zapata and Dufour (1993); García-Chacón et al. (2023)
**Malvidin-3,5-diglucoside**	Anthocyanin	Fruit	LC-MS/MS	Conceição et al. (2020)
**Methionine**	Amino acid	Fruit	Amino-acid composition analysis; review-derived record	Zapata and Dufour (1993); Akter et al. (2011)
**Monounsaturated fatty acids**	Fatty acids	Fruit	GC-FID	Justi et al. (2000)
**Myrciarone A**	Acylphloroglucinol	Peel; seed	Isolation; NMR	Kaneshima et al. (2017); Castro et al. (2020)
**Myrciarone B**	Acylphloroglucinol	Peel; seed	Isolation; NMR	Kaneshima et al. (2017); Castro et al. (2020)

**Myricetin**	Flavonol	Pulp	HPLC-DAD-ESI-MS/MS; UPLC-HR-QTOF-MS/MS	Fracassetti et al. (2013)
**Myricetin 3-O-hexoside**	Polyphenols	Fruit	LC-TOF-MS; HPLC	Fujita et al. (2015); Rodrigues et al. (2020); Fidelis et al. (2020); Conceição et al. (2020)
**Myricetin 3-O-pentoside**	Polyphenols	Fruit	LC-TOF-MS; HPLC	Fujita et al. (2015); Rodrigues et al. (2020); Conceição et al. (2020)
**Myricetin-3-O-β-D-xylopyranoside**	Flavonol glycoside	Extract/product	UHPLC-QTOF-MS/MS	Rodrigues et al. (2020)
**Myricetin-O-deoxyhexoside**	Flavonol glycoside	Peel	LC-MS/MS	Conceição et al. (2020)
**Myricetin-O-hexoside**	Flavonol glycoside	Pulp	HPLC-DAD-ESI-MS/MS; UPLC-HR-QTOF-MS/MS	Fracassetti et al. (2013); Conceição et al. (2020)
**Myricetin-O-pentoside**	Flavonol glycoside	Pulp, peel	HPLC-DAD-ESI-MS/MS; LC-MS/MS	Fracassetti et al. (2013); Conceição et al. (2020)
**Myricitrin**	Flavonol glycoside	Extract/product	UHPLC-QTOF-MS/MS	Rodrigues et al. (2020)
**Neoxanthin**	Carotenoid	Peel	HPLC-PDA; HPLC-PDA-MS	Zanatta and Mercadante (2007)
**Oleic acid**	Fatty acid (ω-9)	Seed	1H-NMR, ^13C-NMR	Filho et al. (2018)
**Oxalic acid**	Organic acid	Fruit	HPLC-DAD	Chirinos et al. (2010)
**p-coumaric**	Phenolic compound	Fruit	LC-MS/MS	Conceição et al. (2020); Fracassetti et al. (2013)
**p-Coumaroyl hexoside**	Hydroxycinnamate con-jugate	Peel	LC-MS/MS	Conceição et al. (2020)
**p-Cymene**	Monoterpene	Fruit	GC-MS	Barroso et al. (2024)
**Pedunculagin**	Ellagitannin	Seed	HPLC-DAD-ESI-MS/MS; UPLC-HR-QTOF-MS/MS	Fracassetti et al. (2013)
**Pelargonidin-3-rhamnoside**	Phenolic compound	Fruit	LC-MS/MS	Conceição et al. (2020)
**Phenylalanine**	Amino acid	Pulp	Amino-acid composi-tion analysis; review-derived record	Akter et al. (2011); Zapata and Dufour (1993)
**Phosphorus (P)**	Mineral	Seed	ICP-OES	Filho et al. (2018)
**Polyunsaturated fatty acid**	Fatty acids	Fruit	LC-MS/MS	Conceição et al. (2020)
**Potassium (K)**	Mineral	Seed	ICP-OES	Filho et al. (2018); Freitas et al. (2019)
**Potentillin**	Ellagitannin	Seed	HPLC-DAD-ESI-MS/MS; UPLC-HR-QTOF-MS/MS	Fracassetti et al. (2013)
**Procyanidin A2**	Proanthocyanidin	Fruit	LC-MS/MS	Conceição et al. (2020)
**Proline**	Amino acid	Pulp	Amino-acid composi-tion analysis; review-derived record	Zapata and Dufour (1993); Akter et al. (2011)
**Pyruvic acid**	Organic acid	Fruit	HPLC-DAD	Chirinos et al. (2010)
**Quercetin**	Flavonol	Pulp	HPLC-DAD-ESI-MS/MS; UPLC-HR-QTOF-MS/MS	Fracassetti et al. (2013); Chirinos et al. (2010)
**Quercetin-3-O-hexoside**	Flavonol glycoside	Pulp; Fruit	HPLC-DAD-ESI-MS/MS; LC-TOF-MS; HPLC	Fracassetti et al. (2013); Fujita et al. (2015); Akter et al. (2011); Cunha-Santos et al. (2022); Rodrigues et al. (2020); Fidelis et al. (2020)
**Quercetin-3-O-pentoside**	Flavonol glycoside	Pulp; Fruit	HPLC-DAD-ESI-MS/MS; LC-TOF-MS; HPLC	Fracassetti et al. (2013); Fujita et al. (2015); Akter et al. (2011); Cunha-Santos et al. (2022); Rodrigues et al. (2020); Fidelis et al. (2020)
**Quercetin-3-O-α-D-arabinofuranoside**	Flavonol glycoside	Extract/product	UHPLC-QTOF-MS/MS	Rodrigues et al. (2020)
**Quercetin-3-rutinoside**	Flavonoid	Fruit	LC-MS/MS	Conceição et al. (2020)

**Rhodomyrtone**	Acylphloroglucinol	Peel; seed	Isolation; NMR	Kaneshima et al. (2017); Castro et al. (2020)
**Rosmarinic acid**	Phenolic acid	Seed coat	HPLC	Fidelis et al. (2018); Conceição et al. (2020)
**Rutin**	Flavonol glycoside	Fruit	HPLC-DAD	Chirinos et al. (2010)
**Salicylic acid**	Organic acid	Fruit	Organic acid analysis (HPLC)	Akter et al. (2011)
**Saturated fatty acids (Tricosanoic acid, Stearic acid, Palmitic acid, Tridecanoic acid and other minor saturated acids such as myristic and lauric acids)**	Fatty acid	Fruit	GC-FID	Justi et al. (2000)
**Serine**	Amino acid	Pulp	Amino-acid composition analysis; review-derived record	Akter et al. (2011); Zapata and Dufour (1993)
**Shikimic acid**	Organic acid	Fruit	Organic acid analysis (HPLC)	Akter et al. (2011)
**Silicon (Si)**	Mineral	Seed	ICP-OES	Filho et al. (2018)
**Sodium (Na)**	Mineral	Seed	ICP-OES	Filho et al. (2018)
**Succinic acid**	Organic acid	Fruit	HPLC-DAD	Chirinos et al. (2010)
**Sucrose**	Carbohydrates	Fruit	Carbohydrate/compositional analysis	Zapata and Dufour (1993); Akter et al. (2011)
**Sulfur (S)**	Mineral	Seed	ICP-OES	Filho et al. (2018)
**Syringic acid**	Phenolic acid	Pulp; seed coat	LC-TOF-MS; HPLC	Fujita et al. (2015); Fidelis et al. (2018); Fidelis et al. (2020)
**Tannins**	Polyphenols	Fruit	HPLC	García-Chacón et al. (2023); Fidelis et al. (2020); Chirinos et al. (2010)
**Tellimagrandin II**	Ellagitannin	Seed	HPLC-DAD-ESI-MS/MS; UPLC-HR-QTOF-MS/MS	Fracassetti et al. (2013)
**Terpinolene**	Monoterpene	Fruit	GC-MS	Quijano and Pino (2007)
**Threonine**	Amino acid	Pulp	Amino-acid composition analysis; review-derived record	Akter et al. (2011); Zapata and Dufour (1993)
**Titanium (Ti)**	Mineral	Seed	ICP-OES	Filho et al. (2018)
**trans-Resveratrol**	Stilbene	Seed/seed coat; Fruit	HPLC; UPLC-ESI-MS/MS	Fidelis et al. (2018); do Carmo et al. (2020); Fidelis et al. (2020); García-Chacón et al. (2023)
**Tricyclene**	Volatile terpene	Fruit	HS-SPME-GC/MS	Souza et al. (2021)
**Triglycerides**	Lipids	Seed oil	$^{1}H/^{13}C$ NMR; oil-composition analysis	Filho et al. (2018)
**Tryptophan**	Amino acid	Fruit	Amino-acid composition analysis; review-derived record	Zapata and Dufour (1993); Akter et al. (2011)
**Tyrosine**	Amino acid	Fruit	Amino-acid composition analysis; review-derived record	Zapata and Dufour (1993); Akter et al. (2011)
**Unsaturated fatty acid**	Lipids	Fruit	GC-FID	Justi et al. (2000)
**Unsaturated ω-3 acyl groups**	Lipids	Fruit	GC-FID	Justi et al. (2000)
**Unsaturated ω-6, Saturated, monounsaturated ω-9 and/or ω-7**	Lipids	Fruit	1H-NMR, 13C-NMR	Filho et al. (2018)
**Valine**	Amino acid	Pulp	Amino-acid composition analysis; review-derived record	Akter et al. (2011); Zapata and Dufour (1993)

**Valoneic acid dilactone**	Hydrolysable tannin derivative	Seed	LC-MS/MS	Conceição et al. (2020)
**Vescalagin**	Ellagitannin	Seed	HPLC-DAD-ESI-MS/MS	Fracassetti et al. (2013); Conceição et al. (2020); Fujita et al. (2015); Fidelis et al. (2020)
**Violaxanthin (all-trans/cis isomers)**	Carotenoid	Peel; Fruit	HPLC-PDA; HPLC-PDA-MS; Amino acid analyzer	Zanatta and Mercadante (2007)
**Zeaxanthin**	Carotenoid	Peel	HPLC-PDA; HPLC-PDA-MS	Zanatta and Mercadante (2007)
**α-Carotene**	Carotenoid	Peel	HPLC-PDA; HPLC-PDA-MS	Zanatta and Mercadante (2007)
**α-fenchene**	Monoterpene	Fruit	GC-MS	Franco and Shibamoto (2000)
**α-Ketoglutaric acid**	Organic acid	Fruit	Organic acid analysis (HPLC)	Akter et al. (2011)
**α-Linolenic acid**	Fatty acid (ω-3)	Seed	1H-NMR, ^13C-NMR	Filho et al. (2018)
**α-phellandrene**	Monoterpene	Fruit	GC-MS	Araújo et al. (2022)
**α-Pinene**	Monoterpene	Fruit	GC-MS	Duke phytochemical database; Conceição et al. (2020)
**α-terpinene**	Monoterpene	Fruit	LC-MS/MS	Conceição et al. (2020)
**β-Carotene**	Carotenoid	Peel	HPLC-PDA; HPLC-PDA-MS	Zanatta and Mercadante (2007)
**β-Caryophyllene**	Sesquiterpene	Fruit	GC-MS	Duke phytochemical database; Conceição et al. (2020)
**β-Cryptoxanthin**	Carotenoid	Peel	HPLC-PDA; HPLC-PDA-MS	Zanatta and Mercadante (2007)
**β-Linalool**	Monoterpene alcohol	Fruit	HS-SPME-GC/MS	Souza et al. (2021)
**β-myrcene**	Monoterpene	Fruit	GC-MS	Barroso et al. (2024)
**β-phellandrene**	Monoterpene	Fruit	GC-MS	Franco and Shibamoto (2000)
**β-Pinene**	Monoterpene	Fruit	GC-MS	Duke phytochemical database; Quijano and Pino (2007)
**γ-terpinene**	Monoterpene	Fruit	GC-MS	Araújo et al. (2022)

Table S2. Solvent systems and annotated ^{1}H NMR coverage.

Solvent system	Major chemical groups represented	Number of annotated records
$CH_3OH:CD_3OD$ (9:1, v/v)	Flavanol derivatives; anthocyanins; ellagic acid; gallic acid; organic acids; fatty acids; amino acids; carbohydrates; other organic compounds	28
$CD_3OD:H_2O$ (1:1, v/v)	Flavanol derivatives; ellagic acid; gallic acid; organic acids; amino acids; carbohydrates; other organic compounds	15
acetone-d_6	Flavanol derivatives; ellagic acid; gallic acid; organic acids; amino acids; carbohydrates; other organic compounds	15
acetonitrile-d_3	Flavanol derivatives; ellagic acid; gallic acid; organic acids; amino acids; fatty acids; carbohydrates; other organic compounds	16
$CDCl_3$	Lipids and fatty acids	10
DMSO-d_6	Ellagic acid; organic acids; amino acids; carbohydrates; other organic compounds	11

$H_2O:D_2O$ (1:1, v/v)	Flavanol derivatives; ellagic acid; gallic acid; organic acids; amino acids; carbohydrates; other organic compounds	14

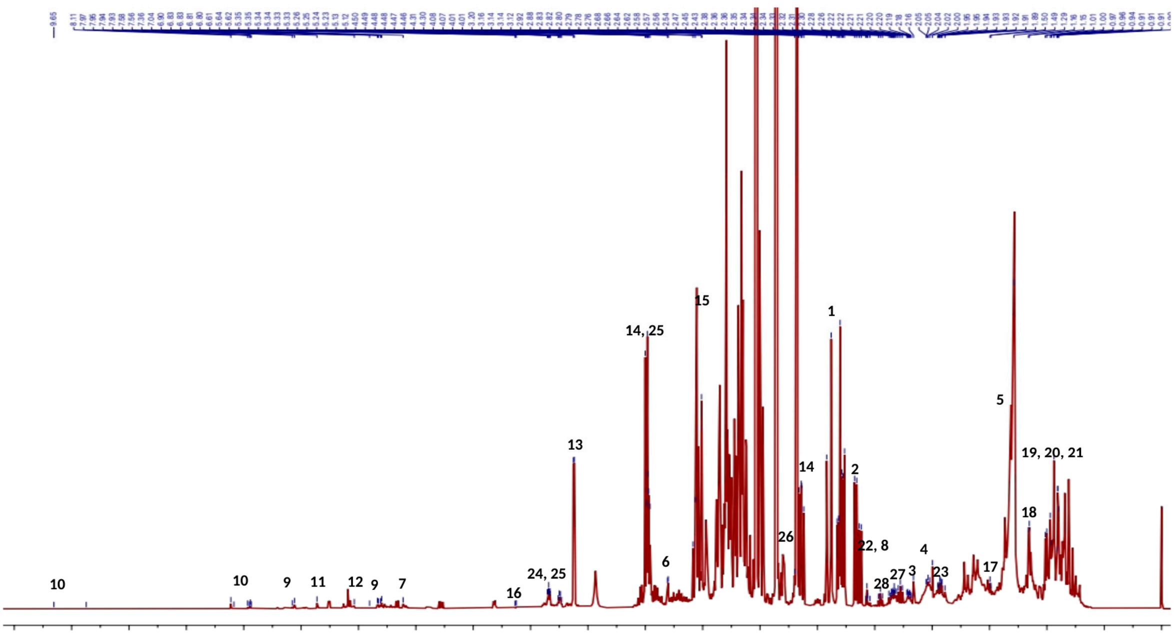

Figure S1. Representative ^{1}H NMR spectrum (400 MHz) of a single extraction replicate of camu-camu (*Myrciaria dubia*) extract in $CH_3OH:CD_3OD$ (9:1, v/v). Characteristic resonances are labeled for selected metabolite annotations. Detailed assignments, multiplicities, and J-coupling constants are provided in Table S3. Chemical shifts are reported in δ (ppm) relative to TMS. Each tissue–solvent condition was analyzed using three independent extraction replicates (n = 3).

Table S3. Solvent-specific ^{1}H NMR chemical-shift features and compound/class annotations in camu-camu (*Myrciaria dubia*) fruit extracts obtained using $CH_3OH:CD_3OD$ (9:1, v/v).

No.	Chemical class	Compound or class annotation	^{1}H NMR chemical shift, δ (ppm)	Multiplicity, J (Hz), integration	Annotation level
1	Organic acids	Citric acid	2.78, 2.90	d, J = 15.7 and 15.6, 1H each	Putative compound annotation
		Malic acid	2.65, 2.80	dd, J = 16.1, 1H each	Putative compound annotation
		Pyruvic acid	2.16	s, 3H	Putative compound annotation
		Acetic acid	2.00	s, 3H	Putative compound annotation
		Lactic acid	1.29	d, J = 6.9, 3H	Putative compound annotation
		Tartaric acid	4.30	d, J = 2.8, 1H	Putative compound annotation
		Fumaric acid	6.61	s, 1H	Putative compound annotation
		Succinic acid	2.54	s, 2H	Putative compound annotation

2	Flavonoid-related compounds	Flavonoid derivatives	6.83, 7.57	d, J = 3.2 and 7.8, 1H each	Class-level annotation
3	Anthocyanins	Cyanidin derivatives	9.65, 8.11,	s; 1H, s; 1H	Class-level annotation
4	Ellagitannins/ellagic-acid-related compounds	Ellagic acid	7.36	s, 1H	Putative compound annotation
5	Hydrolysable phenolics	Gallic acid	7.04	s, 1H	Putative compound annotation
6	Carbohydrates	α-Glucose	5.12	d, J = 3.7, 1H	Putative compound annotation
		β-Glucose	4.49	d, J = 7.8, 1H	Putative compound annotation
		Fructose	4.08	d, J = 7.6, 1H	Putative compound annotation
		Sucrose	5.63	d, J = 4.8, 1H	Putative compound annotation
7	Amino acids	Alanine	1.49	d, J = 7.2, 3H	Putative compound annotation
		Threonine	1.16	d, J = 6.4, 3H	Putative compound annotation
		Valine	1.01	d, J = 7.2, 3H	Putative compound annotation
		Isoleucine	0.95	d, J = 7.5, 3H	Putative compound annotation
		Leucine	0.72-0.76	m, 3H	Putative compound annotation
		Methionine	2.57	t, J = 4.1, 3H	Putative compound annotation
		Glutamine	2.32–2.38; 2.02–2.05	m, 2H and m, 2H	Putative compound annotation
8	Fatty-acyl/lipid compounds	Unsaturated fatty-acyl resonances	5.33	m, 1H	Class-level annotation
		Triglyceride-related resonances	4.47	dd, J = 12.1, 4.0, 2H	Class-level annotation
9	Other organic compounds	Choline	3.20	s, 9H	Putative compound annotation
		Glutamate	2.18–2.22; 1.89–1.97	m, 2H and m, 2H	Putative compound annotation
		γ-Aminobutyric acid (GABA)	2.28; 2.45	t, J = 7.4 and 7.1, 2H each	Putative compound annotation

Note: Chemical shifts are reported as δ (ppm). Annotations are based on diagnostic ^{1}H NMR resonances and comparisons with literature and reference data. Compound-level annotations are considered putative unless supported by orthogonal evidence or authentic standard comparisons. Overlapping fatty acyl signals were reported at the class level. References: [56,58,59,72,87,88].

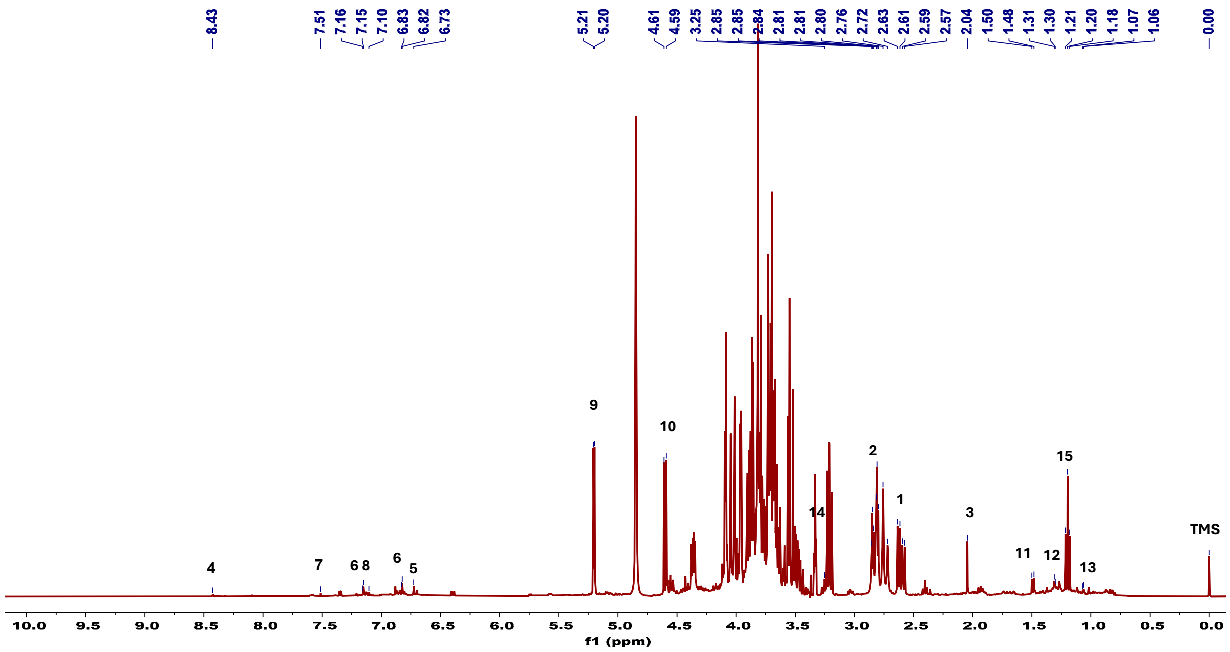

Figure S2. Representative ^1H NMR spectrum (400 MHz) of a single extraction replicate of camu-camu (*Myrciaria dubia*) extract in $CD_3OD:H_2O$ (1:1, v/v). Characteristic resonances are labeled for selected metabolite annotations. Detailed assignments, multiplicities, and J-coupling constants are provided in Table S4. Chemical shifts are reported in δ (ppm) relative to TMS. Each tissue–solvent condition was analyzed using three independent extraction replicates (n = 3).

Table S4. Solvent-specific ^1H NMR chemical shift features and compound/class annotations in camu-camu (*Myrciaria dubia*) fruit extracts obtained using $CD_3OD:H_2O$ (1:1, v/v).

No.	Chemical class	Compound or class annotation	^1H NMR chemical shift, δ (ppm)	Multiplicity, J (Hz), integration	Annotation level
1	Organic acids	Citric acid	2.60	dd, J = 15.9, 7.8, 2H	Putative compound annotation
		Malic acid	2.77	dd, J = 15.6, 4.8, 2H	Putative compound annotation
		Acetic acid	2.04	s, 3H	Putative compound annotation
		Formic acid	8.45	s, 1H	Putative compound annotation
		Fumaric acid	6.73	s, 1H	Putative compound annotation
2	Flavonoid-related compounds	Flavonoid derivatives	6.83, 7.15	d, J = 1.6 and 2.1, 1H each	Class-level annotation
3	Ellagic-acid-related compounds	Ellagic acid	7.51	s, 1H	Putative compound annotation
4	Hydrolysable phenolics	Gallic acid	7.10	s, 1H	Putative compound annotation
5	Carbohydrates	α-Glucose	5.20	d, J = 3.7, 1H	Putative compound annotation
		β-Glucose	4.60	d, J = 7.8, 1H	Putative compound annotation
6	Amino acids	Alanine	1.49	d, J = 7.2, 3H	Putative compound annotation

		Threonine	1.16	d, J = 6.6, 3H	Putative compound annotation
		Isoleucine	1.07	d, J = 7.5, 3H	Putative compound annotation
7	Other organic compounds	Choline	3.20	s, 9H	Putative compound annotation
		Ethanol	1.20	t, J = 7.1, 3H	Putative compound annotation

Note: Chemical shifts are reported as δ (ppm). Annotations are based on diagnostic ^{1}H NMR resonances and comparisons with literature and reference data. Compound-level annotations are considered putative unless supported by orthogonal evidence or authentic standard comparisons. References: [56,58,88].

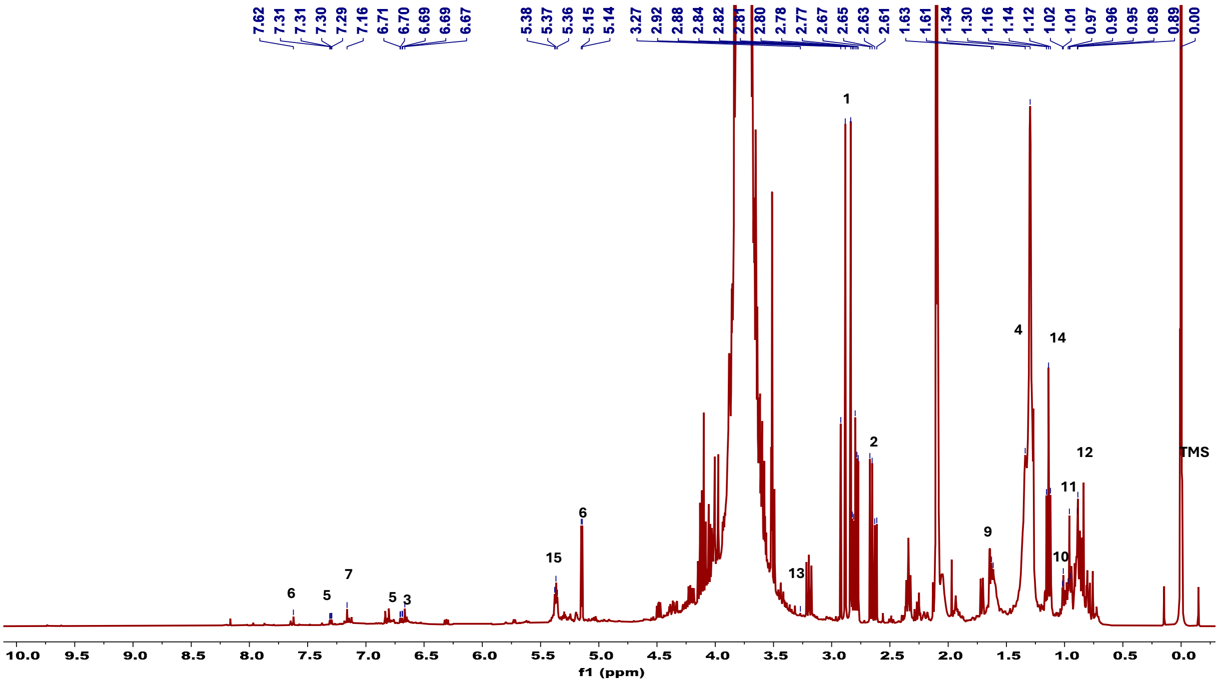

Figure S3. Representative ^{1}H NMR spectrum (400 MHz) of a single extraction replicate of camu-camu (*Myrciaria dubia*) extract in acetone-d$_6$ (CD_3COCD_3). Characteristic resonances are labeled for selected metabolite annotations. Detailed assignments, multiplicities, and J-coupling constants are provided in Table S5. Chemical shifts are reported in δ (ppm) relative to TMS. Each tissue–solvent condition was analyzed using three independent extraction replicates (n = 3).

Table S5. Solvent-specific ^{1}H NMR chemical shift features and compound/class annotations in camu-camu (*Myrciaria dubia*) fruit extracts obtained using acetone-d$_6$.

No.	Chemical class	Compound or class annotation	^{1}H NMR chemical shift, δ (ppm)	Multiplicity, J (Hz), integration	Annotation level
1	Organic acids	Citric acid	2.86	dd, J = 16.5, 15.8, 2H	Putative compound annotation
		Malic acid	2.64	dd, J = 16.1, 7.5, 2H	Putative compound annotation
		Fumaric acid	6.67	s, 1H	Putative compound annotation
		Lactic acid	1.32	d, J = 7.0, 3H	Putative compound annotation
2	Flavonoid-related compounds	Flavonoid derivatives	6.70, 7.30	dd, J = 7.1, 1.7 and 5.1, 1.7, 1H each	Class-level annotation

3	Ellagic-acid-related compounds	Ellagic acid	7.62	s, 1H	Putative compound annotation
4	Hydrolysable phenolics	Gallic acid	7.16	s, 1H	Putative compound annotation
5	Carbohydrates	α-Glucose	5.15	d, J = 3.7, 1H	Putative compound annotation
6	Amino acids	Alanine	1.62	d, J = 7.2, 3H	Putative compound annotation
		Valine	1.01	d, J = 7.0, 3H	Putative compound annotation
		Isoleucine	0.96	t, J = 7.1, 3H	Putative compound annotation
		Leucine	0.89	m, 3H	Putative compound annotation
7	Other organic compounds	Choline	3.27	s, 9H	Putative compound annotation
		Ethanol	1.14	t, J = 7.0, 3H	Putative compound annotation
		Unsaturated fatty-acyl resonances	5.37	t, J = 4.3, 2H	Class-level annotation

Note: Chemical shifts are reported as δ (ppm). The original α-glucose integration value was corrected from 32H to 1H. Assignments are considered putative unless supported by orthogonal evidence or authentic standard comparisons. References: [56,72,87,88].

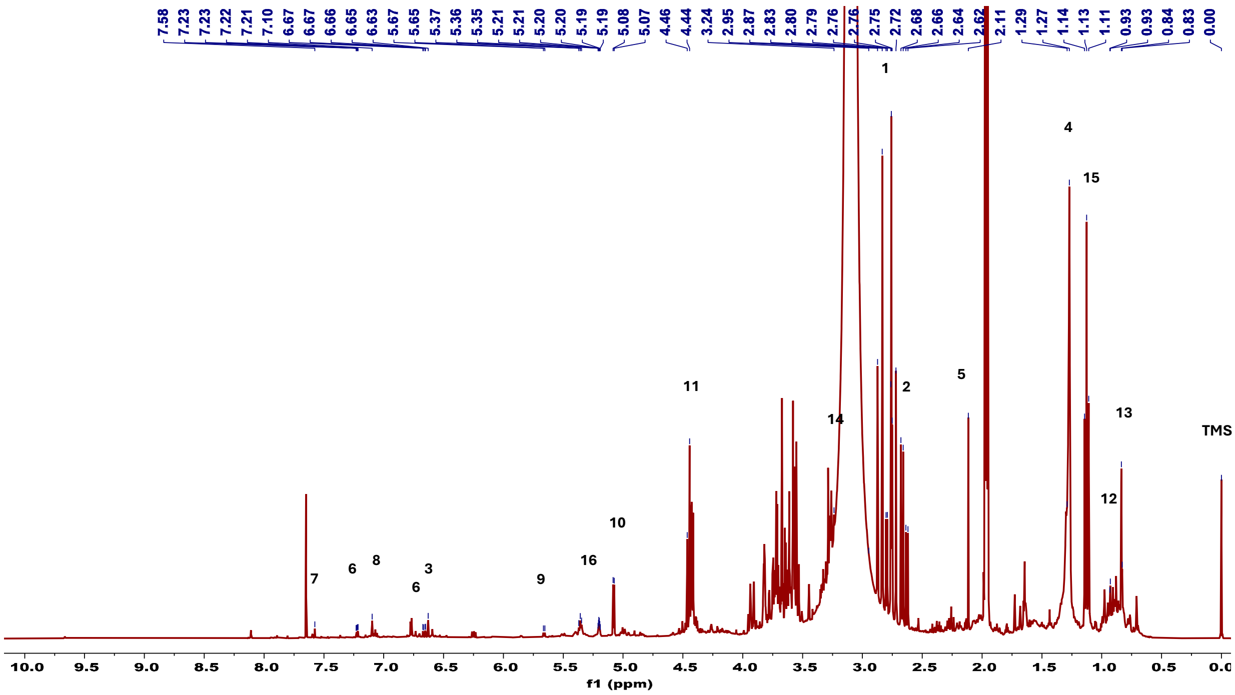

Figure S4. Representative ^{1}H NMR spectrum (400 MHz) of a single extraction replicate of camu-camu (*Myrciaria dubia*) extract in Acetonitrile-d_3 (CD_3CN). Characteristic resonances are labeled for selected metabolite annotations, and detailed assignments, multiplicities, and J-coupling constants are provided in Table S6. Chemical shifts are reported in δ (ppm) relative to TMS. Each tissue–solvent condition was analyzed using three independent extraction replicates (n = 3).

Table S6. Solvent-specific ^{1}H NMR chemical shift features and compound/class annotations in camu-camu (*Myrciaria dubia*) fruit extracts obtained using acetonitrile-d_3.

No.	Chemical class	Compound or class annotation	^1H NMR chemical shift, δ (ppm)	Multiplicity, J (Hz), integration	Annotation level
1	Organic acids	Citric acid	2.80	dd, J = 16.0, 16.0, 2H	Putative compound annotation
		Malic acid	2.65	dd, J = 16.3, 7.2, 2H	Putative compound annotation
		Fumaric acid	6.63	s, 1H	Putative compound annotation
		Lactic acid	1.29	d, J = 7.0, 3H	Putative compound annotation
		Acetic acid	2.11	s, 3H	Putative compound annotation
2	Flavonoid-related compounds	Flavonoid derivatives	6.66, 7.22	dd, J = 7.1, 1.7 and 5.1, 1.6, 1H each	Class-level annotation
3	Ellagic-acid-related compounds	Ellagic acid	7.58	s, 1H	Putative compound annotation
4	Hydrolysable phenolics	Gallic acid	7.10	s, 1H	Putative compound annotation
5	Carbohydrates	Sucrose	5.66	d, J = 4.7, 1H	Putative compound annotation
		α-Glucose	5.08	d, J = 3.7, 1H	Putative compound annotation
		β-Glucose	4.45	d, J = 7.8, 1H	Putative compound annotation
6	Amino acids	Isoleucine	0.93	d, J = 7.2, 3H	Putative compound annotation
		Leucine	0.83	m, 3H	Putative compound annotation
7	Other organic compounds	Choline	3.24	s, 9H	Putative compound annotation
		Ethanol	1.14	t, J = 7.0, 3H	Putative compound annotation
		Unsaturated fatty-acyl resonances	5.36	t, J = 4.5, 2H	Class-level annotation

Note: Chemical shifts are reported as δ (ppm). Specific fatty acid molecular species were not assigned using ^1H NMR data alone. References: [56,72,87,88].

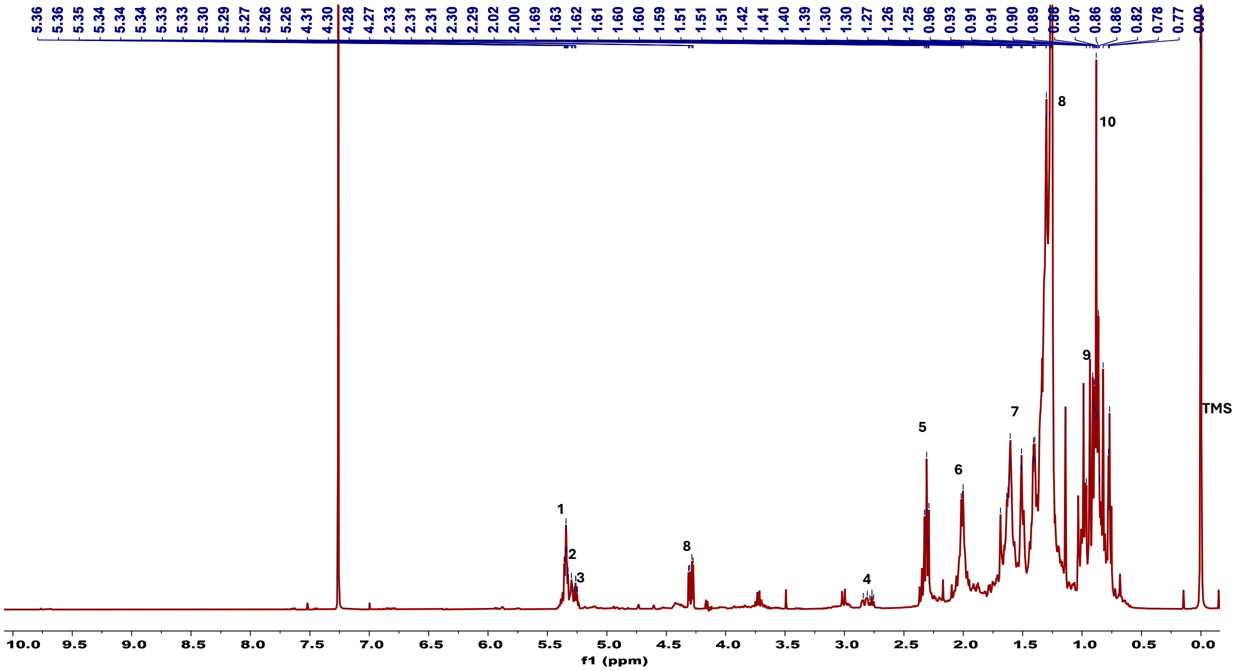

Figure S5. Representative ^1H NMR spectrum (400 MHz) of a single extraction replicate of camu-camu (*Myrciaria dubia*) extract in $CDCl_3$. Characteristic resonances are labeled for selected metabolite annotations, and detailed assignments, multiplicities, and J-coupling constants are provided in Table S7. Chemical shifts are reported in δ (ppm) relative to TMS. Each tissue–solvent condition was analyzed using three independent extraction replicates (n = 3).

Table S7. Solvent-specific ^1H NMR chemical shift regions associated with lipid and fatty acyl structural classes in camu camu (*Myrciaria dubia*) fruit extracts obtained using $CDCl_3$.

No.	Lipid/fatty-acyl annotation	^1H NMR chemical-shift region, δ (ppm)	Proton environment	Annotation level
1	Unsaturated fatty-acyl groups	5.33–5.36	–CH=CH–	Class-level annotation
2	Triglyceride-related resonances	5.29–5.31	Glycerol ester methine region	Class-level annotation
3	Phospholipid-related glycerol resonances	5.25–5.28	Glycerol backbone region	Class-level annotation
4	Polyunsaturated fatty-acyl chains	2.75–2.84	Bis-allylic/allylic methylene region	Class-level annotation
5	Unsaturated fatty-acyl chains	2.29–2.33	Allylic/α-carbonyl methylene region	Class-level annotation
6	Fatty-acyl chains	1.99–2.04	Allylic/α-carbonyl methylene region	Class-level annotation
7	Fatty-acyl chains	1.59–1.69	β-carbonyl/chain methylene region	Class-level annotation
8	Long-chain fatty-acyl methylenes	1.25–1.30	$-(CH_2)n-$	Class-level annotation
9	Fatty-acyl methyl groups	0.93–1.01	Terminal $-CH_3$	Class-level annotation
10	Fatty-acyl methyl groups	0.75–0.91	Terminal $-CH_3$	Class-level annotation

Note: The observed ^1H NMR regions support the presence of lipid and fatty acyl structural classes. Specific fatty acid molecular species, including docosahexaenoic acid and linoleic acid, should not be definitively identified from these ^1H NMR chemical shift regions alone. Molecular species-level

assignment requires orthogonal evidence, such as authentic standard comparison, targeted LC-MS/MS, or additional structural data. Reference: [87,88].

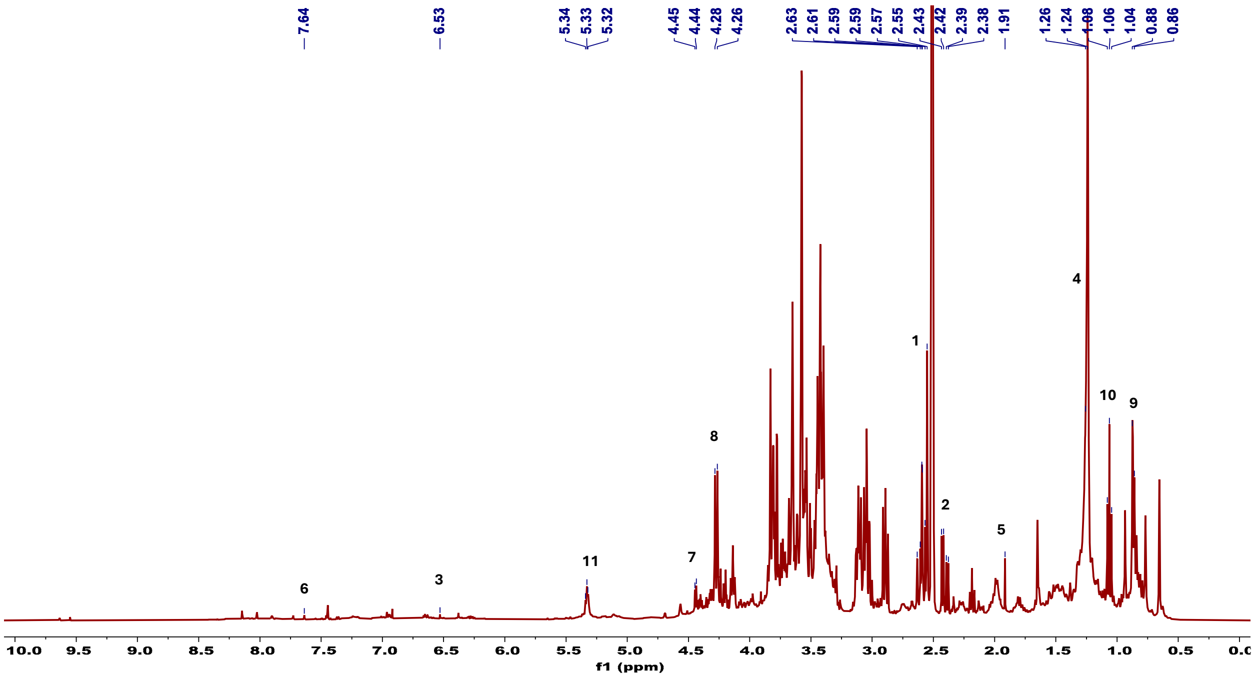

Figure S6. Representative ^{1}H NMR spectrum (400 MHz) of a single extraction replicate of camu-camu (*Myrciaria dubia*) extract in DMSO-d_6. Characteristic resonances are labeled for selected metabolite annotations. The detailed assignments, multiplicities, and J-coupling constants are provided in Table S8. Chemical shifts are reported in δ (ppm) relative to TMS. Each tissue–solvent condition was analyzed using three independent extraction replicates (n = 3).

Table S8. Solvent-specific ^{1}H NMR chemical shift features and compound/class annotations in camu-camu (*Myrciaria dubia*) fruit extracts obtained using DMSO-d_6.

No.	Chemical class	Compound or class annotation	^{1}H NMR chemical shift, δ (ppm)	Multiplicity, J (Hz), integration	Annotation level
1	Organic acids	Citric acid	2.41	dd, J = 15.6, 6.5, 2H	Putative compound annotation
		Malic acid	2.59	dd, J = 9.3, 6.4, 2H	Putative compound annotation
		Fumaric acid	6.53	s, 1H	Putative compound annotation
		Lactic acid	1.25	d, J = 6.7, 3H	Putative compound annotation
		Acetic acid	1.93	s, 3H	Putative compound annotation
2	Ellagic-acid-related compounds	Ellagic acid	7.64	s, 1H	Putative compound annotation
3	Carbohydrates	β-Glucose	4.44	d, J = 4.6, 1H	Putative compound annotation
		Fructose	4.27	d, J = 7.8, 1H	Putative compound annotation
4	Amino acids	Leucine	0.87	d, J = 6.5, 3H	Putative compound annotation

5	Other organic compounds	Ethanol	1.06	t, J = 7.0, 3H	Putative compound annotation
		Unsaturated fatty-acyl resonances	5.33	t, J = 4.0, 2H	Class-level annotation

Note: Chemical shifts are reported as δ (ppm). Specific fatty acid molecular species were not assigned using ^1H NMR data alone. References: [56,87,88].

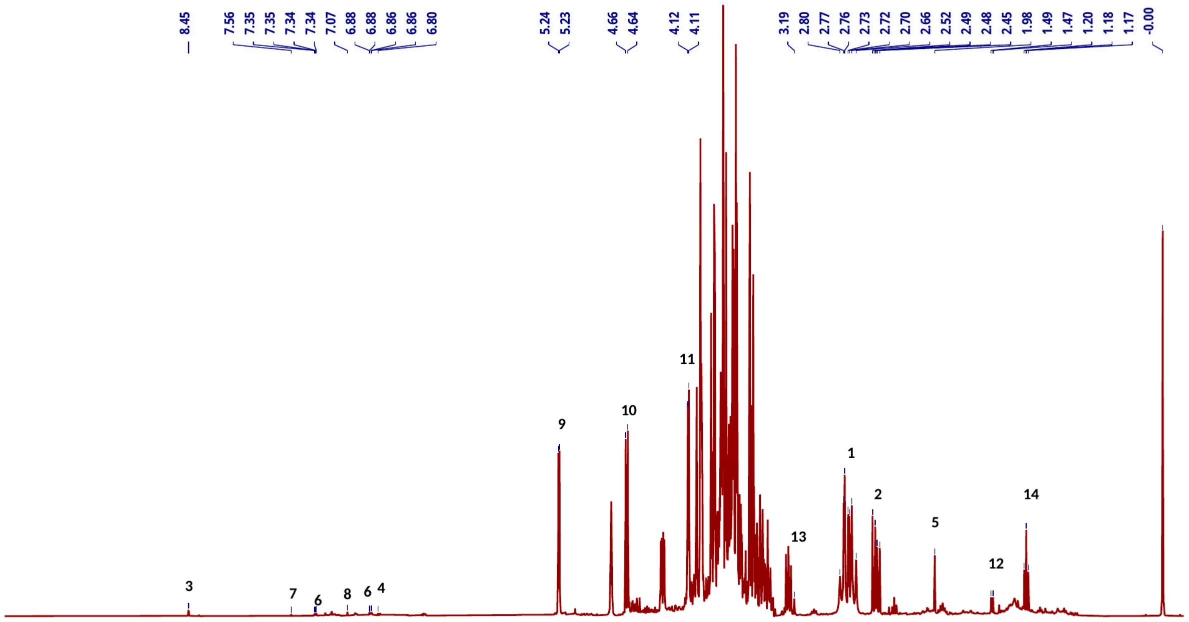

Figure S7. Representative ^1H NMR spectrum (400 MHz) of a single extraction replicate of camu-camu (*Myrciaria dubia*) extract in H_2O:D_2O (1:1, v/v). Characteristic resonances are labeled for selected metabolite annotations, and detailed assignments, multiplicities, and J-coupling constants are provided in Table S9. Chemical shifts are reported in δ (ppm) relative to TMS. Each tissue–solvent condition was analyzed using three independent extraction replicates (n = 3).

Table S9. Solvent-specific ^1H NMR chemical shift features and compound/class annotations in camu-camu (*Myrciaria dubia*) fruit extracts obtained using H_2O:D_2O (1:1, v/v).

**No.**	**Chemical class**	**Compound or class annotation**	**^1H NMR chemical shift, δ (ppm)**	**Multiplicity, J (Hz), integration**	**Annotation level**
1	Organic acids	Citric acid	2.48	dd, J = 15.6, 9.2, 2H	Putative compound annotation
		Malic acid	2.74	dd, J = 15.6, 3.5, 2H	Putative compound annotation
		Formic acid	8.46	s, 1H	Putative compound annotation
		Fumaric acid	6.80	s, 1H	Putative compound annotation
		Acetic acid	1.98	s, 3H	Putative compound annotation
2	Flavonoid-related compounds	Flavonoid derivatives	6.87, 7.35	dd, J = 7.2, 1.7 and 5.0, 1.6, 1H each	Class-level annotation
3	Ellagic-acid-related compounds	Ellagic acid	7.56	s, 1H	Putative compound annotation

4	Hydrolysable phenolics	Gallic acid	7.07	s, 1H	Putative compound annotation
5	Carbohydrates	α-Glucose	5.23	d, J = 3.7, 1H	Putative compound annotation
		β-Glucose	4.65	d, J = 7.9, 1H	Putative compound annotation
		Fructose	4.11	d, J = 7.2, 1H	Putative compound annotation
6	Amino acids	Alanine	1.48	d, J = 7.2, 3H	Putative compound annotation
7	Other organic compounds	Choline	3.19	s, 9H	Putative compound annotation
		Ethanol	1.18	t, J = 7.1, 3H	Putative compound annotation

Note: Chemical shifts are reported as δ (ppm). Assignments were based on diagnostic ^{1}H NMR resonances and comparisons with literature and reference data. Compound-level assignments are considered putative unless supported by orthogonal evidence or authentic standard comparisons. References: [56,58,88].

Table S10. The evidence categories draw on camu-camu biofunctional reviews and metabolite-specific references for choline, GABA, and choline-containing phospholipids [20,61–63].

Compound/class	Biochemical relevance	Evidence basis	Conservative interpretation
Choline	Required for acetylcholine synthesis and phosphatidylcholine metabolism	Nutrition literature [61]	Supports nutritional context only; not proof of clinical efficacy.
GABA	Principal inhibitory neurotransmitter; studied for stress and sleep outcomes	Human supplementation review [62]	Relevant mechanistically; no camu-camu disease claim.
Phosphatidylcholines and lysophosphatidylcholines	Membrane structure, remodeling, and inflammatory signaling	Mechanistic and biomarker literature [63]	Contextual evidence for future mechanistic studies.
Carnitine/acylcarnitines	Fatty-acid transport and mitochondrial energy metabolism	Present LC-MS annotations; targeted confirmation recommended	Hypothesis-generating; targeted quantification and biological testing required.
Spermidine/polyamines	Cellular stress-response and autophagy-related biology	Present LC-MS annotations; targeted confirmation recommended	Hypothesis-generating only; no clinical claim.

Table S11. Reference-checked NMR annotation status table. The table separates putative compound annotations from class-level annotations and unresolved features that require targeted confirmation.

Observed feature	Observed resonance(s)	Reference-check status	Recommended interpretation
Ethanol	δ 1.06-1.20 (t, CH_3; solvent-dependent)	Observed solvent-dependent triplet region; consistent with reference assignment	Putative diagnostic annotation; confirm with complementary resonance and blank/process review
Formic acid	δ 8.45-8.46 (s)	Consistent with reference spectra	Diagnostic annotation
β-Glucose	δ 4.44-4.65 (d, H-1 β-anomer; solvent-dependent)	Consistent with reference spectra	Diagnostic annotation

GABA	δ 2.28 (t, J = 7.4 Hz); δ 2.45 (t, J = 7.1 Hz)	Consistent with reference spectra; overlap possible	Putative diagnostic annotation
Choline	δ 3.19-3.27 (s, $N(CH_3)_3$; solvent-dependent)	Consistent with reference spectra	Diagnostic annotation
Unsaturated fatty-acid/lipid resonances	δ 0.75-1.01, 1.25-1.30, 1.59-1.69, 1.99-2.04, 2.29-2.33, 2.75-2.84, 4.29, 5.25-5.36	Class-level lipid-region pattern; not compound-specific	Class-level annotation; confirm fatty-acid composition by GC-FID/GC-MS or targeted LC-MS/MS
Tartaric acid	δ 4.30 (d, J = 2.8 Hz)	Consistent with reference spectra	Diagnostic annotation

Table S12. Targeted LC-MS/MS analytical conditions, standards, MRM, and quality control parameters.

Analytical component	Method and conditions	Analytical role / validation
Analytical scope	Targeted quantitative reverse-phase LC-MS/MS followed by direct-flow-injection tandem mass spectrometry was used for 12 plant-extract samples in different solvents, screening 209 metabolites.	Quantitative metabolite measurements were used to support metabolite and annotation coverage.
Instrument and software	AB Sciex 4000 QTRAP tandem mass spectrometer coupled to an Agilent 1260 UHPLC; reverse-phase LC followed by direct-flow injection; MRM detection; and Analyst 1.6.2 for project data analysis.	Targeted quantitative analysis was performed using individual chemical calibration standards, corresponding isotope-labeled internal standards, and other internal standards.
Plate controls and standards	Project analytical plate: one blank, three zero samples, seven calibration standard wells, and three quality control wells. The cited analytical method designates seven calibration levels as Cal 1–Cal 7 and uses low-, medium-, and high-concentration quality control materials [70].	Seven calibration standard wells were included in the project plate. Cal 1–Cal 7 are calibration levels in the cited method, not seven metabolite identities.
PITC derivatization	20 μL internal-standard mixture + 10 μL sample extract; dry 30 min under nitrogen; add 50 μL 5% PITC; react 20 min at room temperature; dry 1 h; add 300 μL 5 mM ammonium acetate in methanol; shake 450 rpm for 30 min; centrifuge 50 × g for 5 min.	150 μL extract + 150 μL water for LC-MS/MS; remaining extract + 400 μL DFI buffer for acylcarnitines and C-6 sugars.
Phospholipid preparation	10 μL internal-standard mixture + 5 μL plant extract + 150 μL extraction solution + 600 μL DFI buffer in a deep-well plate.	Direct flow injection MS/MS.

Analytical component	Method and conditions	Analytical role / validation
3-NPH organic-acid derivatization	10 µL internal-standard mixture + 10 µL sample extract + 30 µL 75% aqueous methanol; add 25 µL each of 250 mM 3-NPH, 150 mM EDC, and 7.5% pyridine; shake 450 rpm for 2 h at room temperature; add 350 µL water and 25 µL BHT (2 mg/mL in methanol).	LC-MS/MS analysis of organic acids.
Amino acids and biogenic amines LC-MS/MS	Zorbax Eclipse XDB C18, 3.0 × 100 mm, 3.5 µm, 80 Å, with C18 guard; A = 0.2% formic acid in water, B = 0.2% formic acid in acetonitrile; 100% A at 0–0.5 min, 5% A at 5.5–6.5 min, 100% A at 7.0–9.5 min; 0.5 mL/min; 10 µL; 50 °C; positive ESI sMRM; +5500 V; 500 °C; CUR/GAS1/GAS2 20/40/50; CAD medium; EP +15 V.	Q1, Q3, DP, CE, and CXP were optimized for each analyte and ISTD.
Organic acids LC-MS/MS	Same C18 column; A = 0.01% formic acid in water, B = 0.01% formic acid in acetonitrile; 70% A at 0–1.5 min, 5% A at 8.0 min, 0% A at 8.01–9.5 min, 70% A at 9.6–14.0 min; 0.3 mL/min; 10 µL; 40 °C; negative ESI sMRM; −4500 V; 400 °C; CUR/GAS1/GAS2 20/30/30; CAD medium; EP −10 V.	Q1, Q3, DP, CE, and CXP were optimized for each analyte and ISTD.
Direct-flow-injection MS/MS	Flow: 0.03 mL/min at 0–1.6 min, 0.2 mL/min at 2.4–2.8 min, 0.03 mL/min at 3.0 min; 20 µL injection; positive MRM for phospholipids and acylcarnitines; negative MRM for C-6 sugars; +5500/−4500 V; 200 °C; CUR/GAS1/GAS2 20/40/50; CAD medium; EP/CXP +10/+15 V or −10/−15 V.	The DP, CE, Q11, and Q3 were optimized for each analyte and ISTD.
Calibration and quantification	Cited analytical method [70]: individual chemical standards and corresponding isotope-labeled internal standards from C/D/N Isotopes Inc., Cayman Chemical, Toronto Research Chemicals, Cambridge Isotope Laboratories Inc., Sigma-Aldrich, and IsoSciences; HPHPA and ^{13}C-HPHPA synthesized in-house; analyte-specific stock solutions; Cal 1–Cal 7 prepared in water or 75% aqueous methanol; analyte-to-internal-standard peak-area ratios; quadratic regression with 1/x weighting for amino acids, amino-acid derivatives, biogenic amines, and organic acids; representative single-point calibration with linear regression through zero for classes without individual standards.	Absolute quantification of analytes with individual standards; semi-quantitative analysis of acylcarnitines, C-6 sugars, and phospholipids without individual standards.
MRM transition parameters	Analyte-specific Q1/Q3 precursor/product-ion pairs and associated DP, CE, and CXP settings were optimized for the analytical method [70].	The numerical project-specific Q1/Q3, DP, CE, and CXP values for the 209-metabolite analytical panel were not included in the analytical records supplied to the authors and are therefore not reported.
Quality control and validation	The cited method-validation procedure [70] was as follows: low-, medium-, and high-concentration QC materials were prepared in triplicate; accuracy acceptance was 100 ± 20%; precision CV was < 20%; recovery was generally 80–120%; and carry-over was assessed after Cal 7 versus the following double blank.	Method-validation accuracy, precision, recovery, and carry-over criteria were reported in the cited analytical method.

Analytical component	Method and conditions	Analytical role / validation
Replication and statistical treatment	Three independent extraction replicates for each ^{1}H NMR tissue–solvent condition; targeted DI/LC-MS/MS analysis of 12 plant extract samples. The presence of annotations and record counts were the descriptive endpoints.	Inferential statistical testing was not applied to the annotation-count endpoints.
